# A polypeptide model for toxic aberrant proteins induced by aminoglycoside antibiotics

**DOI:** 10.1371/journal.pone.0258794

**Published:** 2022-04-29

**Authors:** Mangala Tawde, Abdelaziz Bior, Michael Feiss, Feiyue Teng, Paul Freimuth

**Affiliations:** 1 Department of Biological Sciences and Geology, Queensborough Community College, City University of New York, Bayside, New York, United States of America; 2 Department of Natural and Applied Sciences, Cheyney University of Pennsylvania, Cheyney, Pennsylvania, United States of America; 3 Department of Microbiology and Immunology, University of Iowa Carver College of Medicine, Iowa City, Iowa, United States of America; 4 Center for Functional Nanomaterials, Brookhaven National Laboratory, Upton, New York, United States of America; 5 Biology Department, Brookhaven National Laboratory, Upton, New York, United States of America; University of Florida, UNITED STATES

## Abstract

Aminoglycoside antibiotics interfere with the selection of cognate tRNAs during translation, resulting in the synthesis of aberrant proteins that are the ultimate cause of cell death. However, the toxic potential of aberrant proteins and how they avoid degradation by the cell’s protein quality control (QC) machinery are not understood. Here we report that levels of the heat shock (HS) transcription factor σ32 increased sharply following exposure of *Escherichia coli* to the aminoglycoside kanamycin (Kan), suggesting that at least some of the aberrant proteins synthesized in these cells were recognized as substrates by DnaK, a molecular chaperone that regulates the HS response, the major protein QC pathway in bacteria. To further investigate aberrant protein toxic potential and interaction with cell QC factors, we studied an acutely toxic 48-residue polypeptide (ARF48) that is encoded by an alternate reading frame in a plant cDNA. As occurred in cells exposed to Kan, σ32 levels were strongly elevated following ARF48 expression, suggesting that ARF48 was recognized as a substrate by DnaK. Paradoxically, an internal 10-residue region that was tightly bound by DnaK *in vitro* also was required for the ARF48 toxic effect. Despite the increased levels of σ32, levels of several HS proteins were unchanged following ARF48 expression, suggesting that the HS response had been aborted. Nucleoids were condensed and cell permeability increased rapidly following ARF48 expression, together suggesting that ARF48 disrupts DNA-membrane interactions that could be required for efficient gene expression. Our results are consistent with earlier studies showing that aberrant proteins induced by aminoglycoside antibiotics disrupt cell membrane integrity. Insights into the mechanism for this effect could be gained by further study of the ARF48 model system.

## Introduction

Aminoglycoside antibiotics such as kanamycin (Kan) bind to 16S rRNA in the A site of the 30S ribosomal subunit, interfering with the selection of cognate tRNAs during translation [1]. Aberrant proteins are synthesized as a result, including proteins truncated by premature termination or with sequences garbled by frame-shift events or misincorporation of amino acids [2]. By contrast, protein synthesis is completely halted by non-aminoglycoside antibiotics like tetracycline (Tc) [3, 4] and chloramphenicol (Cm) [5]. Importantly, the growth-inhibiting effects of Tc and Cm are reversible, indicating that cells can tolerate absence of new protein synthesis for extended periods, whereas aminoglycosides are bactericidal [6]. Cells can survive exposure to aminoglycosides if they are simultaneously treated with translation-halting antibiotics like Cm however, indicating that the aberrant proteins synthesized in aminoglycoside treated cells are the ultimate cause of the bactericidal effect [6, 7]. Since individual aberrant proteins are synthesized in just one or a few copies per cell, it has not been possible to isolate representative toxic species for mechanistic studies. However, earlier work suggested that the diverse population of aberrant proteins acts in concert to disrupt cell membrane integrity, based on the finding that membrane permeability to ions increases after exposure of cells to aminoglycoside antibiotics [8]. It was suggested that the aberrant proteins might form ion-conducting channels in the membrane [7], but these putative channels have not been isolated and the mechanism for disruption of membrane integrity therefore remains unknown.

For aberrant proteins to have toxic effects they must avoid degradation by the cell’s protein quality control (QC) machinery. The heat shock (HS) response is the major protein QC system in bacteria and consists of a set of chaperones and proteases, together known as HS proteins, that function to eliminate excess unfolded proteins and proteins that are irreparably damaged by exposure to extreme environmental conditions, such as elevated temperature [9]. HS gene expression is closely regulated to enable cells to adapt to changes in the burden of unfolded protein. The HS response is positively regulated by the transcription factor σ32 [10] and negatively regulated by the molecular chaperone DnaK, which binds σ32, promoting σ32 degradation by the FtsH protease [11, 12].

Here we report that σ32 levels increased sharply after exposure of *E*. *coli* cells to the aminoglycoside kanamycin (Kan), indicating that at least some aberrant proteins produced in these cells were recognized as substrates by DnaK. To further investigate aberrant protein toxic potential and interaction with cell QC factors, we studied an acutely toxic aberrant protein (ARF48) that was produced in *E*. *coli* by expression of a cloned gene. Our results support the conclusion that toxic activity and recognition as QC substrates are not mutually exclusive properties of aberrant proteins and further suggest that the QC capacity of cells can be exceeded when aberrant proteins are produced at high rates. Our results are consistent with earlier studies showing that the toxic effect of aberrant proteins induced by aminoglycoside antibiotics results from disruption of cell membrane integrity [8]. Further studies of the ARF48 model system could advance understanding of the mechanism for this toxic effect.

## Materials and methods

### Protein expression constructs

The toxic ARF48 protein is encoded by an open +1 alternate reading frame (ARF) in an *Arabidopsis thaliana* cDNA for a Golgi-localized protein (At3G62370) involved in cell wall synthesis [13], which was obtained from the *Arabidopsis* Biological Resource Center at Ohio State University (clone U10771). A fragment of cDNA coding for the plant protein ectodomain (residues 18–325) was subcloned between the SacII and PstI sites in pASK-IBA3 (www.iba-lifesciences.com) for regulated expression in *E*. *coli* from the vector-encoded TetA promoter. Expression from the TetA promoter is negatively regulated by the Tet repressor, also encoded by pASK-IBA3, and is induced by adding anhydrotetracycline (aTc) to the culture medium. The low concentration of aTc required to bind and inactivate the Tet repressor (0.2 μg/ml) does not inhibit growth of *E*. *coli*. Growth of cells transformed by the ectodomain expression construct was halted immediately following addition of aTc to the culture medium. Deletion studies indicated that an internal region of the ectodomain cDNA fragment was sufficient for the toxic effect. Additional subcloning studies indicated that the toxic factor was a 48-residue polypeptide (ARF48) encoded by an open +1 alternate reading frame in the plant cDNA and that an internal 10-residue region of the ARF48 protein (the KL decapeptide) was required for the toxic effect. Expression constructs for the ARF-NR and ARF-DA variants of ARF48, in which the KL decapeptide coding sequence was substituted by coding sequences for the NR and DA heptapeptides (NRLLLTG and DAGAKAG, respectively), were created by PCR on circular ARF48 expression plasmid template DNA, using divergent-facing primers that annealed to sites flanking the KL decapeptide coding sequence and had 5′ extensions that encoded the NR or DA heptapeptide substitutions, followed by self-ligation of the amplimers.

### Growth analysis

**Fig 1:**
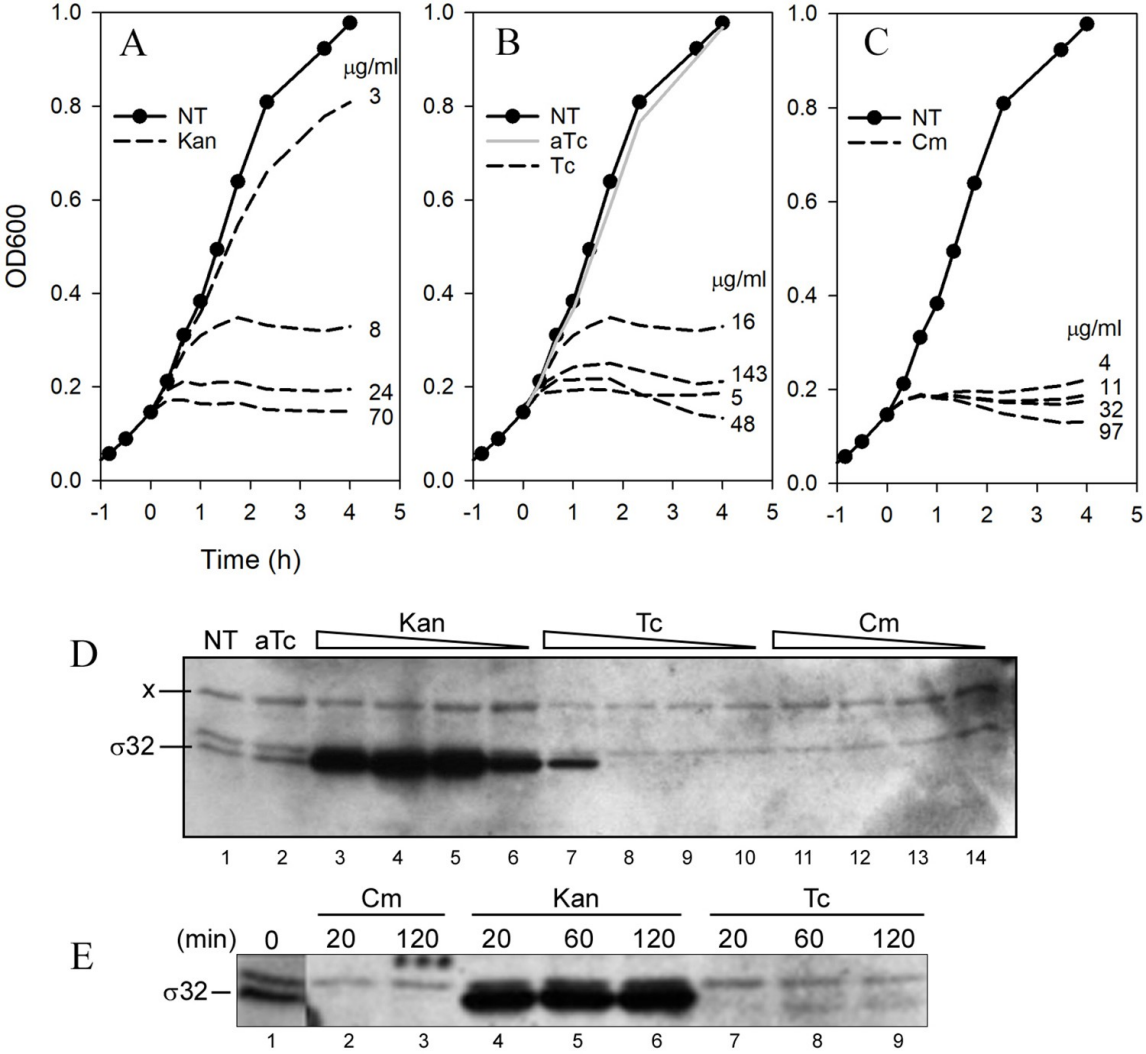
Effect of ribosome-targeting antibiotics on cell growth and σ32 stability. A-C, early log phase cultures of *E*. *coli* BL21 cells were exposed at Time = 0 to the indicated doses of Kan, Tc or Cm (panels A, B and C, respectively), and cell division was then monitored by measuring culture optical density at 600 nm (OD600). Also shown in panel B, cell growth was not inhibited following addition of 0.2 μg/ml anhydrotetracycline (aTc) to the culture medium. D, an equal number of cells was harvested from each culture shown in panels A-C at 60 min after exposure to antibiotics and was analyzed by Western blotting for levels of the σ32 transcription factor. x, an unidentified *E*. *coli* protein that cross-reacted with the antibodies, thus serving as a loading control; NT, lysate from untreated control culture; aTc, lysate from culture exposed to 0.2 μg/ml anhydrotetracycline; triangles above lanes represent increasing doses of antibiotics as shown in panels A-C. E, Western blot analysis of σ32 levels in cells following exposure to the indicated antibiotics for 20, 60, or 120 min as indicated. Cell extract from a non-induced culture was loaded in lane 1.

All growth studies were performed in Luria-Bertani (LB) broth containing Penicillin G (150 μg/ml), using *E*. *coli* BL21 as the host strain except where noted. Frozen stocks of cell strains were first grown overnight at 30°C, without shaking, in 2 ml of LB containing PenG. The starter cultures were then diluted 1:100 in 3.5 ml of LB containing PenG, in plastic 15x90 mm tubes with loose-fitting caps, and the tubes were then tilted about 15–20 degrees from vertical and incubated at 37°C with shaking (300 rpm). Cell growth for all experiments except those shown in Fig 1 was monitored at the indicated times by measuring the optical density of culture samples (diluted 1:10 in saline) at 600 nm, using a Perkin Elmer Lambda 14 UV/VIS spectrophotometer and semi-micro glass cuvettes (1 ml sample volume). For the experiments shown in Fig 1, OD600 measurements were performed on 200 μl of undiluted culture using a 96-well plate reader. Expression of cloned genes from the Tet promoter in pASK-IBA3 was induced in early log phase cultures (OD_600_ ~ 0.35) by addition of 0.2 μg/ml anhydrotetracycline (aTc) to the culture media. To measure cell viability, culture samples were serially diluted in LB broth, mixed with molten top agar and plated on LB agar containing PenG. The number of colonies was then counted after overnight incubation of plates at 37°C.

### Protein gel electrophoresis and Western blotting

Equal volumes of cell cultures were centrifuged, and cell pellets were resuspended directly in 2X Laemmli sample buffer at a concentration of 5 OD_600_ units per ml based on culture OD600 at the time of sampling. To reduce viscosity, samples were sonicated using a cuphorn sonicator or passed through a 27-gauge needle prior to loading on SDS-polyacrylamide gels. Proteins were visualized either by staining gels directly with Coomassie Blue or after semi-dry electroblotting to immobilon membranes for Western blot analysis. Anti-σ32 monoclonal antibodies were purchased from BioLegend, San Diego, CA. Rabbit anti-DnaK and DnaJ serum was kindly provided by B. Bukau. Anti-GroEL antibodies were purchased from Sigma-Aldrich.

### Peptide binding assay

Peptides were synthesized commercially (Peptide 2.0, Inc., Chantilly, VA) with fluorescein-modified N-terminal extensions (FITC-TEKA-) and amidated C-terminal extensions (-KTEQ-NH_2_). The concentrations of peptide solutions were determined by measuring fluorescein absorbance at 485 nm and applying the fluorescein extinction coefficient (50,358/M-cm). 6His-DnaK was purified from the ASKA expression clone [14] by chromatography on Ni-NTA agarose. The concentration of DnaK solutions was determined by BCA assay. 20 nM peptide was incubated with varying concentrations of DnaK in MST buffer (50 mM Tris pH 7.4, 150 mM NaCl, 10 mM MgCl_2_ supplemented with 0.05% Tween-20 and 0.05% bovine serum albumin) for 1 h at 20°C, before analysis by microscale thermophoresis [15], using a NanoTemper Technologies Monolith NT.115 instrument. Plots show the average ± SD for three independent trials for each peptide. Dissociation constants were determined using software provided with the instrument.

### RNA polymerase pull-down assay

An *E*. *coli* strain that produces RNA polymerase containing a 6-histidine-tagged β′ subunit [16] was obtained from M. Kashlev (NIH/NCI) and was transformed with the expression plasmids for ARF48 and the ARF-NR and ARF-DA variant proteins. Cells were grown to early log phase in 50 ml of LB broth containing ampicillin and were then treated with aTc to induce ARF polypeptide expression. At the indicated times 12.5 ml samples of culture were taken and the cells were lysed essentially as described [16]. Briefly, cells were washed in 1 ml of buffer 1 (10 mM Tris pH 8 / 100 mM NaCl / 1 mM β-mercaptoethanol / 5% glycerol), disrupted by sonicating 2x 15s with a microprobe in 300 μl of buffer 1 and then centrifuged 5min in a microcentrifuge. Protein concentration in supernatants was determined by BCA assay, and 100 μg samples of each lysate were then adjusted to 750 mg/ml by dilution with buffer 1. Samples were then adjusted to 0.05% Tween20 and 1 mM imidazole and incubated for 1 h on ice with 20 μl of Super Ni-NTA beads (Protein Ark, UK), with frequent mixing. Beads were pelleted by centrifugation for 1 min at 830x g in a swinging bucket rotor, the unbound fraction (supernatant) was saved, and the beads were then washed twice in 100 μl buffer 1 containing 10 mM imidazole, then once in 100 μl buffer 1 containing 40 mM glycine. Bound proteins were then eluted in 50 μl of buffer 1 containing 150 mM imidazole.

Proteins in 15 μl samples of the pull-down and flow-through fractions were then run on 7% polyacrylamide gels and either stained with Coomassie Blue or transferred to PVDF membranes for Western blot analysis. Membranes were blocked for > 1h in PBS-T containing 5% BSA and then probed for 1 h with antibodies diluted in PBS-T containing 0.5% BSA. Mouse monoclonal antibodies against *E*. *coli* σ70 and σ32 were purchased from BioLegend (SanDiego, CA). Rabbit anti-GroEL IgG was purchased from Sigma-Aldrich. Rabbit sera against *E*. *coli* DnaK and DnaJ proteins was kindly provided by B. Bukau.

### Microscopy

Culture samples (0.3 ml) collected at the indicated times were centrifuged and the cell pellets were resuspended in 20–100 μl of Tris-buffered saline pH 8 (TBS) and held on ice. Cell suspensions were then stained with 1 μl of Hoechst 33342 (10 mg/ml stock solution was first diluted 1:100 in TBS). After incubation for 10 min on ice, 2 μl samples of cell suspension were mounted on coverslides using agarose blocks as described [17]. Cells were then examined by epi-fluorescence microscopy, using a Zeiss Axiophot and a 63X oil immersion lens. Exposure times for 2-channel images (both transmitted and reflected light) were set automatically using on-board Zen 2011 software. In some experiments as noted, exposure times for reflected light images were fixed at 1 s, for comparison of relative fluorescence intensity. Brightfield images of *E*. *coli* cell aggregates were taken with a Nikon Eclipse T2 inverted microscope using a 20X lens and laser-based LED white light source, located at the Brookhaven National Laboratory Center for Functional Nanomaterials.

## Results

### Aberrant proteins produced in aminoglycoside-treated *E*. *coli* are substrates for DnaK

In contrast to ribosome-targeting antibiotics that inhibit translation initiation or elongation, aminoglycosides do not block protein synthesis but instead interfere with selection of cognate aminoacyl-tRNAs, resulting in the synthesis of aberrant proteins [2]. Since each aberrant protein is produced in just one or a few copies per cell, it has not been possible to isolate and characterize representative species. Nevertheless, the diverse population of aberrant proteins appears to act in concert, causing rapid halting of cell growth and loss of cell viability [6]. While the precise mechanism remains unknown, increased cell permeability following exposure to aminoglycosides led to the hypothesis that toxic aberrant proteins target the cell membrane, perhaps forming channels that disrupt membrane integrity [7]. This mechanism would require that toxic aberrant proteins avoid degradation by the cell’s protein quality control (QC) machinery, which normally eliminates aberrant protein species by proteolysis [9].

The molecular chaperone DnaK plays a key role in protein QC by binding reversibly to exposed hydrophobic regions in unfolded proteins, blocking non-specific interactions that could disrupt protein folding [18]. DnaK also binds the heat shock (HS) transcription factor σ32 [11], but in contrast to the positive effect on most substrate proteins, interaction with DnaK promotes degradation of σ32 by proteolysis [19]. DnaK-dependent proteolysis of σ32 is competitively inhibited during periods of increased protein synthesis or exposure to harsh environmental conditions that damage existing proteins, resulting in transient accumulation of σ32 protein and activation of the HS response [20]. We therefore monitored σ32 concentration to determine whether aberrant proteins synthesized in aminoglycoside-treated cells were recognized as substrates by DnaK.

Early log phase *E*. *coli* cells were exposed to the aminoglycoside kanamycin (Kan), or to the non-aminoglycoside antibiotics tetracycline (Tc) and chloramphenicol (Cm). Cell division was halted soon after exposure to all three antibiotics over a range of concentrations, as shown by leveling of the growth curves (Fig 1, panels A-C). Cell viability decreased by 5–6 orders of magnitude during the first hour of exposure to Kan but was fully retained during exposure to Cm (S1 Fig). σ32 levels in cells after 20, 60 or 120 min of antibiotic exposure were then determined by Western blotting. σ32 accumulated to high relative concentration in cells exposed to Kan, even at a low dose (3 μg/ml) that slowed but did not completely halt cell division, whereas σ32 was not detected in cells exposed to Cm or to any but the highest concentration (143 μg/ml) of Tc (Fig 1D; only the 60 min time point is shown). Importantly, in contrast to the transient increase in σ32 concentration that normally occurs during an effective HS response, σ32 concentration remained elevated in cells exposed to Kan (Fig 1E). At least some aberrant proteins synthesized in Kan-treated cells therefore appeared to be recognized as substrates by DnaK, raising the question whether toxic species might correspond to a subset of aberrant proteins that escapes recognition or resists degradation by QC factors. Furthermore, although σ32 was strongly elevated in Kan-treated cells, the ensuing cell death indicates that the HS response, which normally promotes cell survival to proteotoxic stress, was not effective. This could result from the error-prone mode of protein synthesis induced by Kan, which would block synthesis of functional HS proteins required to effectively promote cell survival.

In contrast to the elevated levels of σ32 in cells exposed to Kan, σ32 was not detected in cells exposed to Tc or Cm (Fig 1E). In normal cells, σ32 is synthesized constitutively and steady state levels therefore reflect the rate of σ32 degradation by the FtsH protease [12, 21, 22]. Consistent with this mechanism, the absence of detectable σ32 in cells treated with Cm or Tc indicates that synthesis of σ32 has been blocked and that residual σ32 protein has been degraded. The persistence of σ32 at elevated levels in Kan-treated cells therefore suggests that degradation of residual σ32 was blocked. Taken together, our results suggest that DnaK may be titrated by excess aberrant proteins synthesized in Kan-treated cells and thus unavailable to bind σ32 and promote σ32 degradation by FtsH.

### A gene-encoded toxic aberrant protein

**Fig 2:**
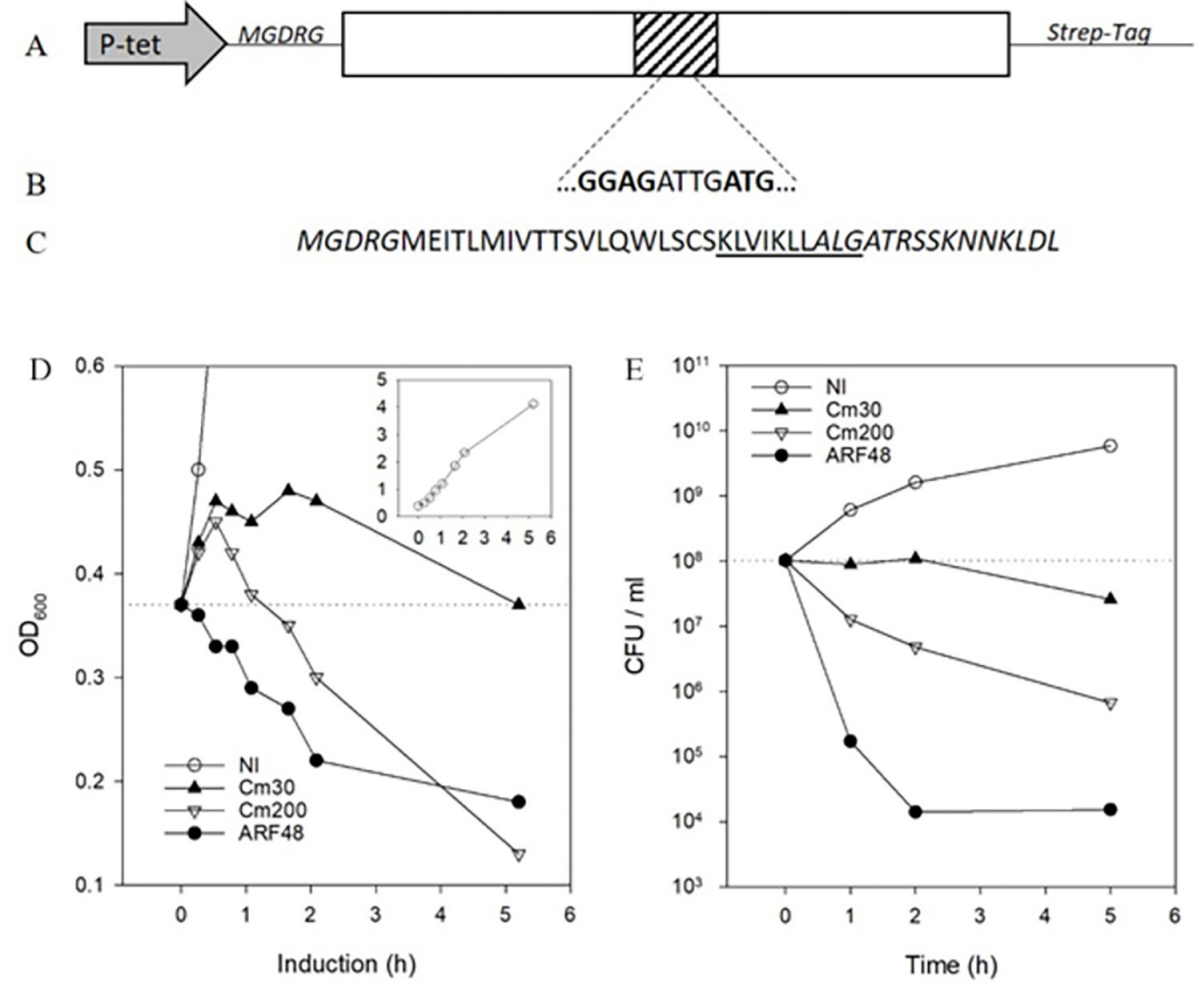
An alternate reading frame-encoded polypeptide is toxic when expressed in *E*. *coli*. A, an *Arabidopsis thaliana* cDNA (Genbank accession At3G62370; open rectangle) that was acutely toxic when expressed in *E*. *coli* from the inducible Tet promoter (P-tet arrow) in pASK-IBA3. Deletion studies showed that an internal region of cDNA (shaded box, not drawn to scale) was sufficient for the toxic effect. B, a Shine-Dalgarno-like sequence [23] contained within the toxic region of cDNA. The ATG initiation codon for this putative translation start site is positioned in the +1 alternate reading frame (ARF) relative to the cDNA-encoded plant protein. C, the ARF-encoded 48-residue protein (ARF48) produced when the coding sequence was translated from the start site in pASK-IBA3. A 10-residue sequence (KL decapeptide) that was tightly bound by DnaK *in vitro* is underlined. Vector-encoded residues are shown in italic type. D, the effect of ARF48 expression or treatment with 30 or 200 μg/ml Cm on growth of *E*. *coli* BL21 cells. ARF48 expression was induced by adding anhydrotetracycline (0.2 μg/ml) to the culture medium. NI, a non-induced culture grown in parallel; inset, growth of the NI control culture. E, the effect of ARF48 expression or treatment with Cm on cell viability. Samples of culture taken at 0, 1, 2 and 5 h after ARF48 induction or treatment with Cm were serially diluted and plated on LB agar. Colonies were counted after incubating the plates overnight at 37°C.

The difficulty in isolating representative toxic aberrant proteins from aminoglycoside-treated cultures has limited understanding of the mechanisms of toxic activity. To circumvent this problem, we studied an aberrant protein, ARF48, that has an acutely toxic effect when expressed in *E*. *coli* from a cloned gene. ARF48 is encoded by an alternate reading frame in an *Arabidopsis thaliana* cDNA clone (Fig 2A), which we discovered was translated in *E*. *coli* from an internal position in the mRNA resembling a Shine-Dalgarno site (Fig 2B). For the studies described here, the ARF48 coding sequence was subcloned behind the inducible TetA promoter and bona fide translation start site in expression plasmid pASK-IBA3. The TetA promoter is rapidly activated following addition of the inducer anhydrotetracycline (aTc) to the culture medium, resulting in production of the 48-residue protein shown in Fig 2C. Cell division was halted immediately following induction of ARF48 expression (Fig 2D) and cell viability decreased about 10,000-fold by 2 h after induction (Fig 2E). ARF48 expression therefore mimics the bactericidal effect of aminoglycoside antibiotics [6]. By contrast, cells remained viable for several hours following growth arrest by 30 μg/ml of the bacteriostatic antibiotic Cm (Fig 2E). At higher concentration, Cm was increasingly toxic (Fig 2E).

### ARF48 overexpression is required for its acutely toxic effect

**Fig 3:**
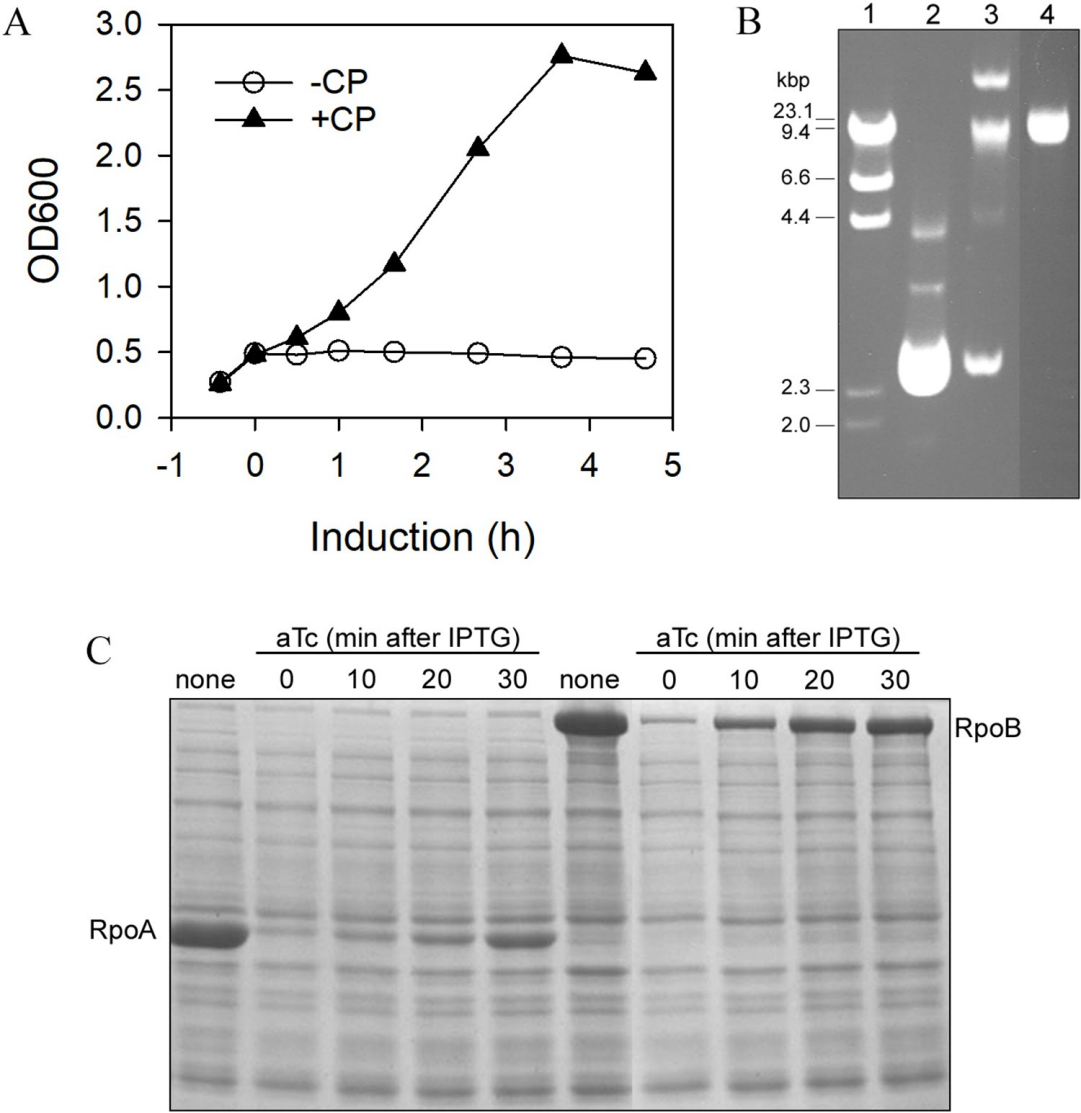
Effect of co-resident plasmids on the ARF48 toxic effect. A, cell growth following ARF48 induction (at T = 0) in *E*. *coli* BL21(DE3) cells transformed by pARF48 alone (-CP, open circles) or in co-transformed cells containing pARF48 and a co-resident plasmid pVS10-Sp^R^, which confers resistance to spectinomycin (+CP, solid triangles). Both pARF48 and pVS10_Sp^R^ are based on ColE1 replicons and therefore belong to the same plasmid incompatibility group. B, agarose gel electrophoresis of plasmid DNA (uncut) recovered from cells that were co-transformed by pARF48 and pVS10_Sp^R^ and grown in the presence of both selecting antibiotics (ampicillin and spectinomycin), was loaded in lane 3. Purified samples of pARF48 and pVS10_Sp^R^ DNA (uncut) were loaded in lanes 2 and 4, respectively, and migrated at the expected sizes (3.2 kb and 15.2 kb, respectively). Phage lambda DNA digested with HindIII was loaded in lane 1, and the sizes of restriction fragments is indicated at left. C, *E*. *coli* BL21DE3 cells co-transformed by pARF48 and pACYC-pET-based expression plasmids for either RpoA or RpoB, which belong to a different plasmid incompatibility group, were grown to early log phase and then treated with Isopropyl β-D-1-thiogalactopyranoside (IPTG) at time 0 to induce expression of RpoA or RpoB proteins (*E*. *coli* RNA polymerase α and β subunits, respectively). After incubation for 0, 10, 20 or 30 min, samples of each culture were treated with aTc to induce ARF48 expression. After further incubation for 2 h, an equal number of cells from each culture was lysed and the lysates were electrophoresed in an SDS-polyacrylamide gel which was then stained with Coomassie blue. Samples from cultures not treated with aTc were loaded in lanes 1 and 6. The positions of RpoA and RpoB proteins in the gel are indicated. The corresponding growth curves for this experiment are shown in S3 Fig.

Proteins expressed from genes cloned in multi-copy plasmid vectors, such as the ColE1-based ARF48 expression plasmid (pARF48), often are produced at high rates, leading to rapid accumulation of the proteins to high concentration [24]. To determine whether ARF48 overexpression was required for the acutely toxic effect, *E*. *coli* BL21 cells hosting pARF48 were transformed with a second ColE1-based plasmid (pVS10_Sp^R^), which carries a selectable gene conferring resistance to the antibiotic spectinomycin. Growth of the co-transformed cells in the presence of both selecting antibiotics (ampicillin and spectinomycin) was expected to reduce the copy number of both plasmids by about 50%, since the total number of ColE1 replicons is maintained at around 15 per cell [25]. The toxic effect indeed was attenuated when ARF48 was expressed in the co-transformed cells (Fig 3A). DNA gel electrophoresis confirmed that cells contained approximately equal amounts of each co-resident plasmid (Fig 3B).

These results suggest that the rate of ARF48 production varies in proportion to the gene copy number, and that the acutely toxic effect is manifested only when the rate of production exceeds the QC capacity to degrade ARF48. However, the pVS10_Sp^R^ plasmid copy number also is reduced by about 50% in the co-transformed cells, and it is therefore possible that levels of the plasmid-encoded spectinomycin resistance factor might not be sufficient to completely inactivate the antibiotic. If spectinomycin activates the HS response, then residual amounts of the antibiotic might prime the co-transformed cells for enhanced degradation of ARF48, possibly accounting for the observed suppression of the toxic effect. We therefore studied whether σ32 levels increase after exposure of non-resistant cells to spectinomycin. As shown in S2 Fig, σ32 levels were unchanged following exposure to spectinomycin, indicating that in contrast to Kan, spectinomycin does not induce the synthesis of aberrant proteins that activate the HS response. This is consistent with earlier studies showing that growth inhibition by spectinomycin is reversible [26]. Together these results suggest that spectinomycin has no significant role in suppression of the ARF48 toxic effect in co-transformed cells.

In contrast to these results, the toxic effect was not attenuated when ARF48 was expressed in cells co-transformed by plasmids from a different incompatibility group (S3 Fig). This was expected since the two plasmid replicons (ColE1 and p15A) are regulated independently, and the pARF48 plasmid copy number therefore should not be reduced [25]. The coresident plasmids in this experiment were p15A replicons and carried genes coding for the *E*. *coli* RpoA or RpoB proteins (RNA polymerase α and β subunits) cloned behind the IPTG-inducible T7/lac promoter in the pACYCDuet expression vector (Novagen). Interestingly, in addition to halting cell growth, continued synthesis of the RpoA and RpoB proteins also was halted following induction of ARF48 expression in the co-transformed cells (Fig 3C). To determine whether the block in protein synthesis following ARF48 expression was restricted to proteins encoded by plasmid-borne genes or also extended to proteins encoded by genes on the cell chromosome, we studied the effect of ARF48 expression on IPTG-inducible synthesis of β-galactosidase, which is encoded by the single-copy *lacZ* gene located on the chromosome. As shown in Table 1, expression of ARF48 for just 5 min was sufficient to effectively block subsequent expression of LacZ in response to IPTG. ARF48 overexpression therefore results in rapid, global inhibition of gene expression.

**Table 1:** ARF48 blocks expression of β-galactosidase.

Hours after IPTG	+ARF48^a^	-ARF48^b^
1	3.0 ± 17%	472 ± 22%
2	13.8 ± 16%	1137 ± 7%

Cells were treated with aTc to induce expression of ARF48 or were mock-treated, and then were treated 5 min later with IPTG to induce expression of β-galactosidase. After culturing for an additional 1 or 2 h, the amount of β-galactosidase enzyme activity (Miller units) in extracts of equal numbers of cells from each culture was determined.

^a^ β-galactosidase activity (Miller units) in cells expressing ARF48

^b^ β-galactosidase activity (Miller units) in control cells not expressing ARF48

### ARF48 is recognized as a substrate by DnaK

**Fig 4:**
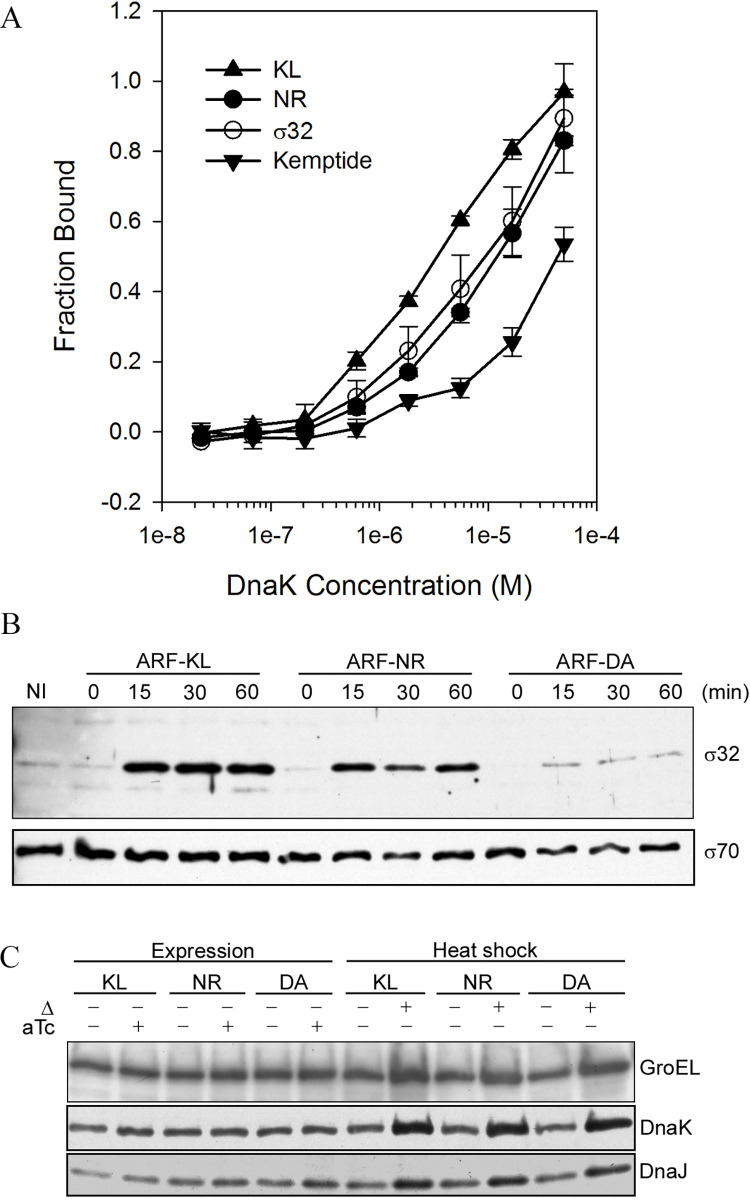
ARF48 is a substrate for DnaK *in vitro* and *in vivo*. A, equilibrium binding of fluorescein-labeled synthetic peptides (20 nM) to increasing concentrations of purified *E*. *coli* DnaK protein, determined by microscale thermophoresis [15]. All synthetic peptides were flanked by the same N- and C-terminal extensions (FITC-TEKA- and -KTEQ, respectively). KL, FITC-TEKAKLVIKLLALGKTEQ; NR, FITC-TEKANRLLLTGKTEQ; σ32, FITC-TEKAQRKLFFNLRKTEQ; Kemptide, FITC-TEKALRASLGKTEQ. Earlier studies showed that the NR and σ32 peptides were bound by DnaK *in vitro* [28, 30], and that Kemptide [31] was poorly recognized by comparison [32]. The mean +/- standard deviation for three independent assays of peptide binding at each concentration of DnaK are shown. B, relative levels of σ32 protein in BL21 cells at 0, 15, 30 or 60 min after induction of ARF48 (ARF-KL) or the ARF-NR and ARF-DA variants, determined by Western blotting. NI, a non-induced control culture. Levels of the major sigma factor σ70 were determined in parallel (lower panel). The results shown are representative of three independent experiments. C, heat shock protein levels in cells before and after expression of ARF48 and the ARF-NR and ARF-DA variant proteins (lanes 1–6) or after heat shock of non-induced cultures at 42°C for 15 min (lanes 7–12).

The copy number dependence of the toxic effect suggests that the cell’s QC capacity to eliminate ARF48 might be exceeded when ARF48 is overexpressed. To study whether ARF48 indeed is recognized as a QC substrate, we first analyzed the ARF48 amino acid sequence for potential binding sites for DnaK, using the program *Limbo* [27]. *Limbo* predicted two partially overlapping binding sites for DnaK contained within ARF48 residues 25–34 (KLVIKLLALG; hereafter the KL decapeptide; underlined in Fig 2C). Purified DnaK indeed bound synthetic KL decapeptide tightly *in vitro*, with about 3-fold higher affinity than the canonical DnaK peptide substrate NRLLLTG [28, 29], as determined by microscale thermophoresis [15] (Fig 4A). Furthermore, the toxic effect was attenuated or abrogated, respectively, when ARF48 was modified to substitute the KL decapeptide sequence with the NRLLLTG or the DAGAKAG heptapeptide sequences, to produce the ARF-NR and ARF-DA variants (S4A Fig). The KL decapeptide therefore not only was recognized by DnaK *in vitro* but also was required for the ARF48 toxic effect.

To determine whether ARF48 also was recognized as a substrate by DnaK *in vivo*, we monitored whether σ32 levels changed following ARF48 expression. As in Kan-treated cells, σ32 levels increased following ARF48 expression and remained elevated (Fig 4B). σ32 levels also increased following expression of ARF-NR but were unchanged following expression of ARF-DA (Fig 4B). These results therefore suggest that ARF48 and ARF-NR were recognized as substrates by DnaK *in vivo*. To determine whether DnaK was required for the toxic effect, ARF48 and the ARF-NR and ARF-DA variants were overexpressed in an *E*. *coli* mutant that lacks functional DnaK [33]. As seen in cells containing wild-type DnaK, growth of the DnaK-null mutant cells was halted abruptly following ARF48 expression and was slowed following expression of the ARF-NR variant but was unaffected by expression of ARF-DA (S4B Fig). DnaK itself therefore was not required for the ARF48 toxic effect. Together these results support the model that the QC machinery capacity to process aberrant proteins is exceeded when ARF48 is overexpressed, and further suggest that the KL decapeptide mediates ARF48 interaction with some other factor(s), other than DnaK, that leads to cell death.

### ARF48 triggers an abortive HS response

Stabilization of σ32 usually indicates that HS gene expression has been activated [10], normally resulting in increased production of HS proteins that promote cell survival during periods of proteotoxic stress. However, the loss of cell viability following ARF48 expression (Fig 2E) indicates that the HS response was not effective in these cells despite the elevated levels of σ32. To further investigate the HS response in these cells, we monitored levels of several HS proteins, including DnaK, DnaJ and GroEL. Levels of these HS proteins changed very little following ARF48 induction (Fig 4C). By contrast, levels of these same HS proteins increased when non-induced cultures were shifted to 45°C for 10 min (Fig 4C), thus indicating that the HS response was functional in these cells prior to ARF48 expression. Taken together, these results suggested that some step in the HS response downstream of σ32 stabilization must be blocked following ARF48 induction.

**Fig 5:**
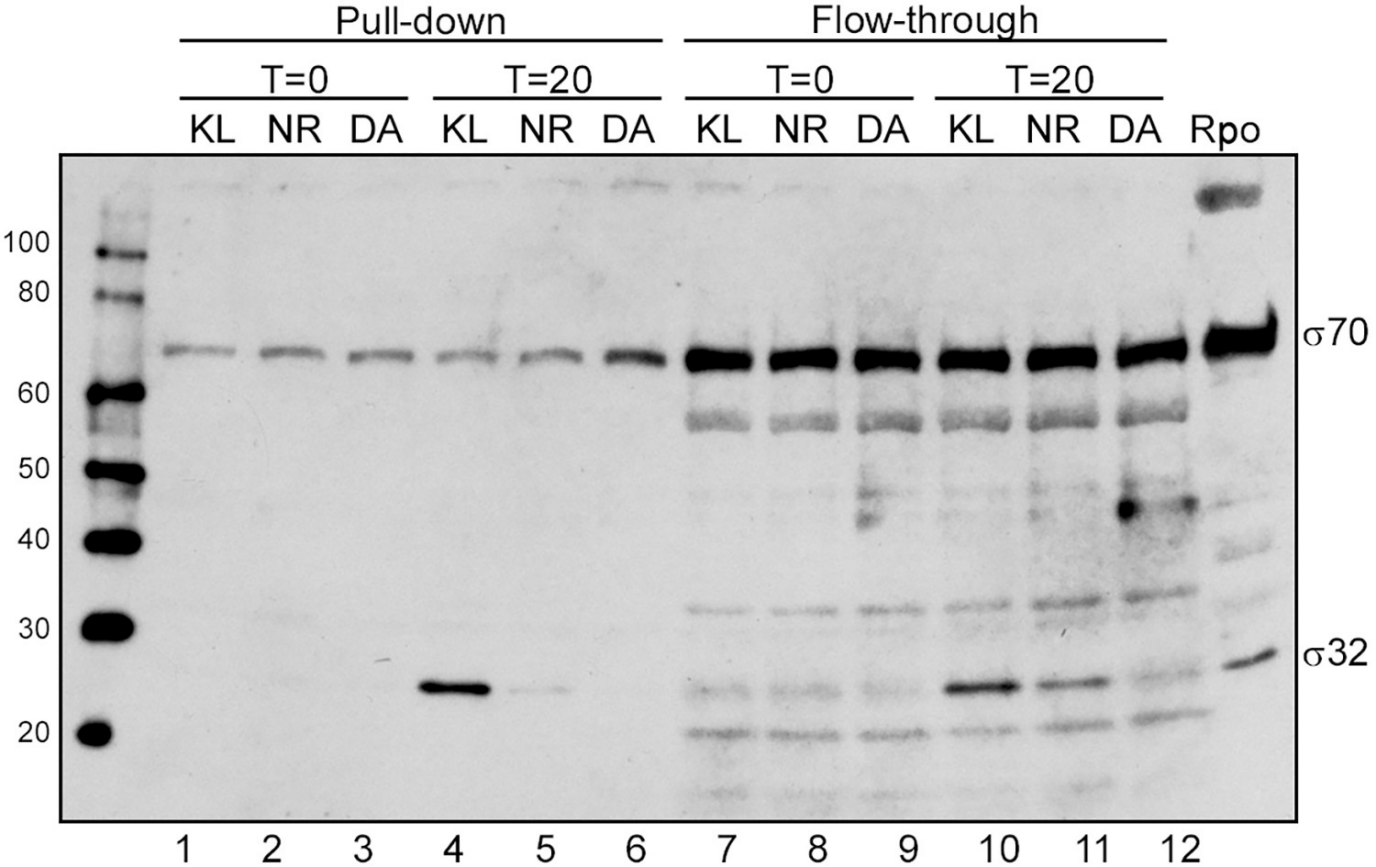
Excess σ32 that accumulates following ARF48 induction associates with RNA polymerase. ARF48 and the ARF-NR and ARF-DA variant polypeptides were expressed in an *E*. *coli* strain that produces RNA polymerase containing a 6-histidine-tagged β′ subunit [34]. Samples of culture were collected immediately before induction (T = 0) and 20 min after ARF protein induction (T = 20), and equal amounts of protein (100 μg) were incubated with Ni-NTA beads as indicated in the Methods section. Samples of the bound (Pull-down) and unbound (Flow-through) fractions were then electrophoresed in an SDS-polyacrylamide gel, transferred to an immobilon membrane, and then probed with antibodies specific for σ70 and σ32. An identical set of samples run in parallel on a separate gel that was then stained with Coomassie blue showed that equal amounts of RNA polymerase (RpoB and RpoC subunits) were present in each sample (S5 Fig).

To investigate the functional status of the σ32 protein that accumulated following ARF48 expression, we asked whether the protein could form stable complexes with RNA polymerase. To study this, ARF48 was expressed in an *E*. *coli* strain that produces RNA polymerase (RNAP) containing a 6-histidine-tagged βʹ subunit [16]. As occurred in cells containing normal untagged RNAP, growth of the strain producing 6-His-tagged RNAP halted rapidly following addition of aTc to the culture medium to induce ARF48 expression, and σ32 levels were elevated in these cells. Following ARF48 induction, RNAP was purified from cell lysates by metal affinity chromatography, and the sigma factors associated with the purified enzyme were then analyzed by Western blotting. RNAP isolated from cells expressing ARF48 was associated with an increased amount of σ32 protein (E-σ32) in comparison to cells expressing ARF-NR or ARF-DA (Fig 5), suggesting that the σ32 protein that accumulates following ARF48 expression is functionally intact. The failure of cells to increase production of HS proteins following ARF48 expression therefore likely results from a block either in transcription of HS genes by E-σ32 or in translation of HS protein mRNAs by cell ribosomes.

### ARF48 induces nucleoid condensation

**Fig 6:**
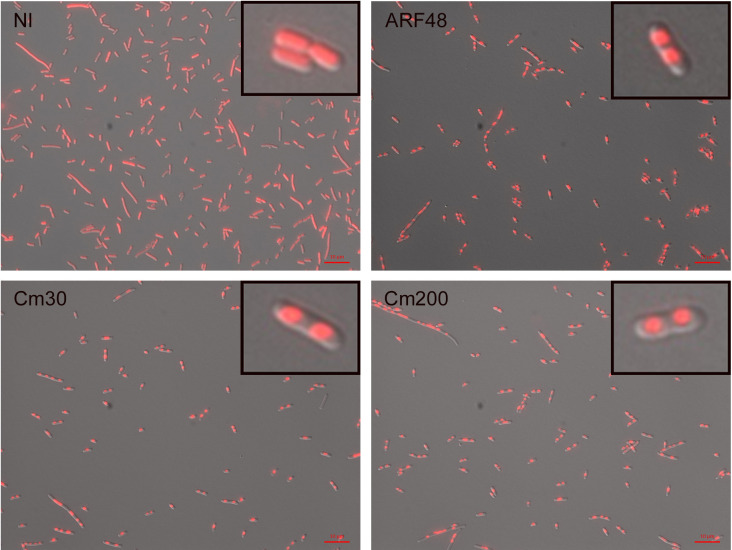
ARF48 increases cell uptake of Hoechst 33342 and induces radial condensation of nucleoids. An early log phase culture of BL21 cells transformed by pARF48 was divided and treated either with anhydrotetracycline (aTc; 0.2 μg/ml) to induce ARF48 expression or with 30 or 200 μg/ml Cm. At 10 min after ARF48 induction or 60 min after treatment with Cm, cell samples were stained with H33342 and examined by fluorescence microscopy. Exposure times were automatically set by camera software and were 0.22 s for ARF48 and Cm samples and 1.7 s for a sample of non-induced (NI) culture grown in parallel. Scale bar, 10 μm. Results shown are representative of multiple independent experiments.

The culture OD600 following ARF48 expression typically decreased by about 30% (Fig 2D), possibly indicative of cell lysis. The cultures never cleared completely as would occur after infection by a lytic phage however, and cell morphology in these cultures appeared normal by light microscopy. Furthermore, samples of culture medium did not contain levels of protein detectable by gel electrophoresis, indicating that cell lysis was not extensive. To further investigate cell membrane integrity, we studied uptake of the DNA-binding fluorescent dye Hoechst 33342 (H33342) by fluorescence microscopy. Following ARF48 induction, cells accumulated approximately 8-times more H33342 than non-induced cells, based on the exposure times required to produce images of equal fluorescence intensity (Fig 6), indicating that cell permeability to H33342 increases following ARF48 expression. Interestingly, the microscopy also showed that nucleoids became radially condensed by 5–10 min after induction of ARF48 expression, whereas the nucleoids in non-induced cells had a radially expanded conformation (Fig 6).

Cm has long been known to cause nucleoids to become radially condensed [35], an effect thought to result from disruption of transertion, the coupled transcription and co-translational insertion of inner membrane (IM) proteins into the cell membrane [36, 37]. During transertion, the cognate IM protein genes are anchored to the cell membrane [38], thus stretching the nucleoid into an open or radially expanded conformation [39]. Consistent with earlier studies, we found that nucleoids became radially condensed following exposure of BL21 cells to Cm (Fig 6; Cm30 and Cm200), but the effect took longer to develop in comparison to the rapid condensation of nucleoids following ARF48 expression and was not uniformly apparent in all cells until 30–45 min after exposure. As occurred following ARF48 expression, uptake of H33342 also was enhanced following exposure to Cm. In contrast to the bactericidal effect of ARF48 however, cells remain viable during several hours of exposure to 30 μg/ml Cm (Fig 2D), therefore indicating that the changes in membrane permeability and nucleoid conformation are reversible in these cells. Exposure to Kan also caused nucleoids to become radially condensed, but in contrast to Cm the effect was only transient and by 1 h after exposure nucleoids again appeared radially expanded (S6 Fig). Interestingly, transient condensation of nucleoids also occurs when transcription is inhibited by rifampicin, and the re-expansion of nucleoids in that case is thought to result from invasion of the condensed nucleoid by 30S and 50S ribosomal subunits, which accumulate in rifampicin-treated cells [40]. A similar mechanism might account for the re-expansion of nucleoids in Kan-treated cells because aminoglycosides do not completely halt translation [2] and ribosomal subunits therefore should be present continuously to penetrate the nucleoid.

**Fig 7:**
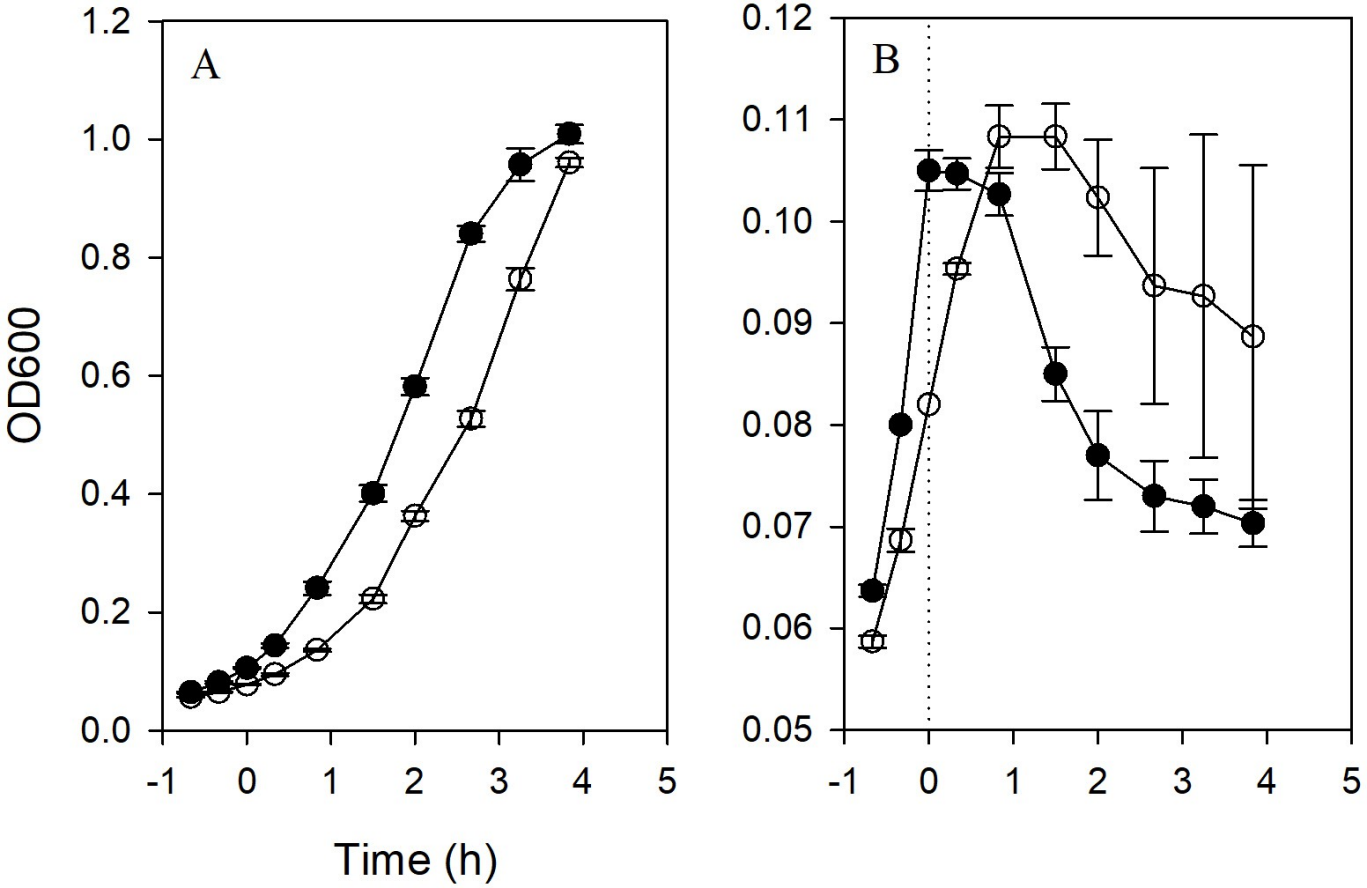
The ARF48 toxic effect is enhanced in medium containing NaCl. *E*. *coli* BL21 cells transformed by pARF48 were grown in standard LB broth, which contains 0.17 M NaCl (filled circles), or in modified LB that lacked NaCl (open circles). A, growth rates of non-induced cultures; B, growth rates of cultures treated with aTc at early log phase to induce expression of ARF48. The experiment was performed with triplicate cultures for each condition, and the mean +/- standard deviation of the OD600 measurements at each time point were plotted.

Membrane-associated proteases in *Pseudomonas aeruginosa* promote resistance to aminoglycoside antibiotics by degrading aberrant proteins that are synthesized by antibiotic-compromised ribosomes [41]. Consistent with the notion that the toxic effect of these aberrant proteins results from disruption of cell membrane integrity [7], *Pseudomonas* protease mutants were found to exhibit sensitivity to NaCl, probably resulting from increased uptake of sodium ions [41]. Since *E*. *coli* cells expressing ARF48 exhibited enhanced uptake of H33342, we wondered if they also might exhibit sensitivity to NaCl. To test this, BL21 cells transformed with the ARF48 expression plasmid were grown in standard LB medium, which contains 0.17 M NaCl, or in modified LB lacking NaCl. In non-induced control cultures, the cell growth rate was slightly faster in standard LB containing NaCl than in NaCl-free LB (Fig 7A). In induced cultures by contrast, the presence of NaCl in the growth medium enhanced the ARF48 acutely toxic effect, accelerating both the onset of cell growth arrest, as measured by culture OD600, and the subsequent decrease in culture OD600 (Fig 7B). These results support the conclusion that ARF48 disrupts the IM permeability barrier.

Interestingly, cells from both induced cultures (+/- NaCl) tended to aggregate when mounted under standard glass cover-slides in buffer containing salt (PBS), as shown by brightfield microscopy (S7 Fig). However, cell aggregation was not observed or was much reduced when salt was omitted from the mounting medium or when agarose pads were used in place of glass cover-slides to limit diffusion of cells mounted in salt-containing medium [17]. Together these results suggest that the increased membrane permeability resulting from ARF48 expression also is manifested at the cell surface, causing exposure of hydrophobic substances that promote cell aggregation that is enhanced by salt. Cell aggregation thus might account for the decreasing culture OD600 that characteristically occurs following ARF48 expression in standard medium containing salt.

## Discussion

Misfolded and aberrant proteins have toxic potential [6, 42] and are the ultimate cause of cell death by aminoglycoside antibiotics, but the underlying mechanisms have proven difficult to elucidate. Furthermore, it is not understood how aberrant proteins escape degradation by the cell’s protein QC machinery to exert their toxic effects. A complicating factor is that exposure to aminoglycoside antibiotics results in synthesis of a highly diverse group of aberrant proteins, each probably synthesized in just one copy per cell [7], which has precluded direct isolation and study of individual aberrant protein species. Here we showed that levels of the HS transcription factor σ32 increased markedly following treatment of *E*. *coli* with the aminoglycoside Kan. The simplest explanation is that at least some of the diverse aberrant proteins synthesized in these cells were recognized as substrates by the molecular chaperone DnaK [20], thus disrupting DnaK-dependent degradation of σ32 by the membrane-associated FtsH protease. The question remained however whether all aberrant proteins were degraded with equal efficiency by the QC machinery, or whether toxic species might represent a subset of aberrant proteins that are relatively resistant to degradation. Stabilization of σ32 usually results in activation of HS gene expression, leading to increased levels of HS proteins that promote cell survival by eliminating excess unfolded and aberrant proteins. Once protein homeostasis has been restored, the normal regulation of σ32 stability by DnaK and FtsH also is restored and is reflected by a decrease in σ32 protein levels. The finding that σ32 levels increased and then remained elevated following exposure to Kan therefore suggests that the HS response in these cells was blocked at some stage downstream of σ32 stabilization.

To investigate the abortive HS response in more detail, we studied an aberrant protein, ARF48, that mimicked the bactericidal effect of aminoglycosides when expressed in *E*. *coli* from a cloned gene. ARF48 proved to be an excellent model for this study, since it not only was bactericidal but also caused σ32 levels to increase and remain elevated, as occurred following exposure to Kan. Knowledge of the ARF48 amino acid sequence and access to the gene enabled us to discover a 10-residue region, the KL decapeptide, that not only strongly interacted with DnaK *in vitro*, but also was required for both the acutely toxic effect and for stabilization of σ32 at elevated levels. Toxic activity and recognition by the QC machinery therefore are not mutually exclusive properties of aberrant proteins, at least in the case of ARF48. How then was ARF48 able to exert its toxic effect? Our finding that toxic activity was attenuated when the ARF48 expression plasmid copy number was reduced suggests that the QC machinery capacity to process aberrant proteins might be exceeded when aberrant proteins are produced at high rates, as can occur when aberrant proteins are overexpressed from genes cloned on multi-copy plasmid vectors or when aminoglycoside antibiotics are used above the minimum inhibitory concentration.

In contrast to the strong increase in σ32 levels, there was little change in the levels of several different HS proteins, including DnaK, DnaJ and GroEL, following ARF48 expression. This suggests that ARF48 interfered with some stage of the HS response downstream of σ32 stabilization. HS gene expression, and gene expression in general, likely were inhibited by the radial condensation of nucleoids that occurred rapidly following ARF48 expression. Nucleoids in growing cells have an expanded conformation thought to result from coupled transcription and co-translational insertion (transertion) of inner membrane (IM) proteins into the cell membrane [36, 37, 39]. Based on modeling studies [37], it was proposed that the expanded nucleoid conformation promotes recycling of the 30S and 50S ribosomal subunits back into the genome for efficient coupling of transcription and translation.

Nucleoids become radially condensed when transcription or translation are inhibited by antibiotics [36, 37], but it is unlikely that either of these essential processes are specifically inhibited by ARF48. Interestingly, nucleoid condensation was accompanied by increased cell permeability to the fluorescent dye H33342 as well as to salt, indicating that the IM permeability barrier is compromised following expression of ARF48. Taken together, these results suggest that ARF48 might disrupt an IM-associated function that is required for transertion. Earlier studies suggested that aberrant proteins synthesized in aminoglycoside-treated cells might form channels in the IM, thus accounting for the increased cell permeability to toxic extracellular ions [7]. Whether the aberrant proteins themselves form *de novo* channels or alternatively disrupt the normal function of pre-existing IM channels remains an open question that could be resolved by further study of the ARF48 model system. The absence of an N-terminal signal peptide suggests that ARF48 is unlikely to target the cell membrane via SecYEG translocons. However, the SecA translocase is an alternate route by which ARF48 might access the membrane since recent studies, reviewed in [43], have shown that SecA alone can form ring-pore structures capable of conducting ions as well as proteins across the membrane, the latter by a signal peptide-independent mechanism.

## Supporting information

S1 Fig Bactericidal effect of Kanamycin. Samples of an *E*. *coli* BL21 culture growing in LB broth were treated at early log phase (T = 0) with Cm (30 μg/ml) or Kan (50 μg/ml). After further incubation at 37°C for 30 or 60 min, samples of the untreated (UT) and antibiotic-treated cultures were diluted (serial 1:10), and 3 μl of each dilution was spotted onto an LB agar plate. The plate was then photographed after incubation overnight at 37°C.
(TIF)

S2 Fig Spectinomycin does not activate the HS response. A, a culture of BL21 cells transformed by pARF48 was divided at early log phase and treated with either aTc (0.2 μg/ml) to induce ARF48 expression, or with Cm, Kan, Tc or Spectinomycin (Spct) at 30, 25, 50 or 20 μg/ml, respectively. An aliquot of culture also was left untreated (NI) for comparison. Cultures were then incubated at 37°C with shaking, and cell growth was monitored by measuring culture optical density at 600 nm (OD600) using a microplate reader. B, samples of each culture were collected at 20, 60 and 90 min after treatment, and cells were lysed in 2X SDS-PAGE sample buffer at a concentration of 1.2 OD600 units per ml. Equal volumes of each sample were then analyzed by Western blotting, using antibodies against σ32 and σ70. Only the 20 min and 60 min samples of the Spectinomycin-treated culture were analyzed.
(TIF)

S3 Fig The ARF48 acutely toxic effect is not attenuated in cells co-transformed by pARF48 and plasmids from a different incompatibility group. *E*. *coli* BL21DE3 cells were co-transformed with pARF48, a ColE1 replicon, and pACYCDuet-RpoA or pACYCDuet-RpoB, which are p15A-based replicons from a different plasmid incompatibility group that contain genes coding for the *E*. *coli* RpoA and RpoB proteins cloned behind the IPTG-inducible T7/lac promoter. Early log phase cultures of the co-transformants were treated with IPTG at time 0 to induce expression of RpoA or RpoB (panels A and B, respectively), and cell growth was then monitored by measuring culture OD600 (solid lines with open circles). At 0, 10, 20 or 30 min later, samples of culture were removed and treated with aTc to induce ARF48 expression (numbered arrows). Growth of the aTc-treated cultures also was monitored as above (dotted lines with filled symbols).
(TIF)

S4 Fig DnaK is not required for the ARF48 toxic effect. A, growth of *E*. *coli* BL21 cells (which contain wild-type DnaK) following expression of ARF48 or the ARF-NR and ARF-DA variants. △, non-induced control; ▲, ARF48; ○, ARF-NR; ●, ARF-DA. B, same experiment as A, but proteins were expressed in a mutant *E*. *coli* strain that lacks the DnaK gene (*ΔdnaK*) [33]. The results shown are representative of 3 independent experiments.
(TIF)

S5 Fig Analysis of protein content in bound and unbound fractions of RNA polymerase pull-down assay. An exact duplicate SDS-polyacrylamide gel of the Western blot shown in Fig 5 was stained with Coomassie blue. A sample of purified RNA polymerase was loaded in the last lane (labeled rpo). Bands corresponding to the RNA polymerase α, β, β′, and σ70 subunits are labeled (A, B/C, and D, respectively).
(TIF)

S6 Fig Nucleoids are only transiently condensed in cells treated with Kan. A culture of *E*. *coli* BL21 cells growing in LB broth at 37°C was divided and treated with either Cm (30 μg/ml) or Kan (50 μg/ml) at early log phase. A culture sample also was left untreated (UT) for comparison. Cell samples were then collected after further incubation for 20, 40 or 60 min, washed with PBS, stained with H33342, and mounted on glass slides under agarose pads [17] for fluorescence microscopy using a 40X oil immersion lens. The exposure time for each image was automatically set by microscope software. Scale bar = 20 μm.
(TIF)

S7 Fig ARF48 expression increases cell propensity to aggregate. ARF48 expression was induced at early log phase in *E*. *coli* BL21 cultures growing either in standard LB broth, which contains 0.17 M NaCl or in modified LB broth lacking NaCl (see Fig 7). 2 h after induction, cell samples from induced and non-induced cultures were washed in PBS, fixed in 4% paraformaldehyde, and then examined by brightfield microscopy. When cell suspensions were mounted under standard glass coverslides, cells from both induced cultures formed cell aggregates (arrows), whereas cells from non-induced cultures did not aggregate. By contrast, when cell suspensions were mounted under agarose pads, which blocks diffusion of cells on the microscope slide, cell aggregation was not observed. Similarly, cell aggregation was not observed when cells from induced cultures were suspended in salt-free buffer (10 mM phosphate pH 7.4) and mounted under standard glass coverslides.
(TIF)

S1 Raw images (PDF)
