# Supplementary figures and images for: A polypeptide model for toxic aberrant proteins induced by aminoglycoside antibiotics
Source: PLoS One. 2022 Apr 29;17(4):e0258794. doi: 10.1371/journal.pone.0258794 (PMC9053816; doi:10.1371/journal.pone.0258794)

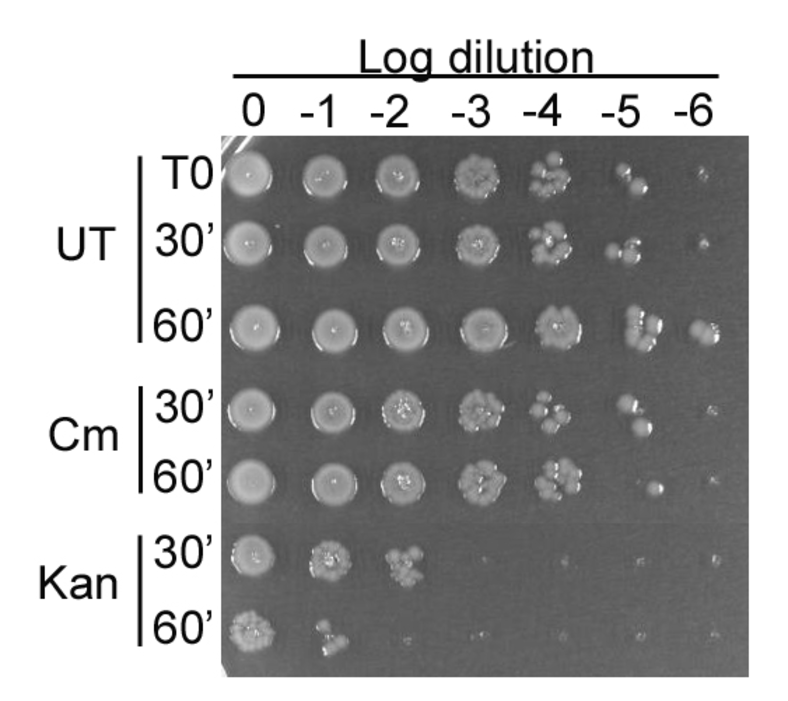

Supplement: S1 Fig — Samples of an E. coli BL21 culture growing in LB broth were treated at early log phase (T = 0) with Cm (30 μg/ml) or Kan (50 μg/ml). After further incubation at 37°C for 30 or 60 min, samples of the untreated (UT) and antibiotic-treated cultures were diluted (serial 1:10), and 3 μl of each dilution was spotted onto an LB agar plate. The plate was then photographed after incubation overnight at 37°C. (TIF) [file pone.0258794.s001.tif]

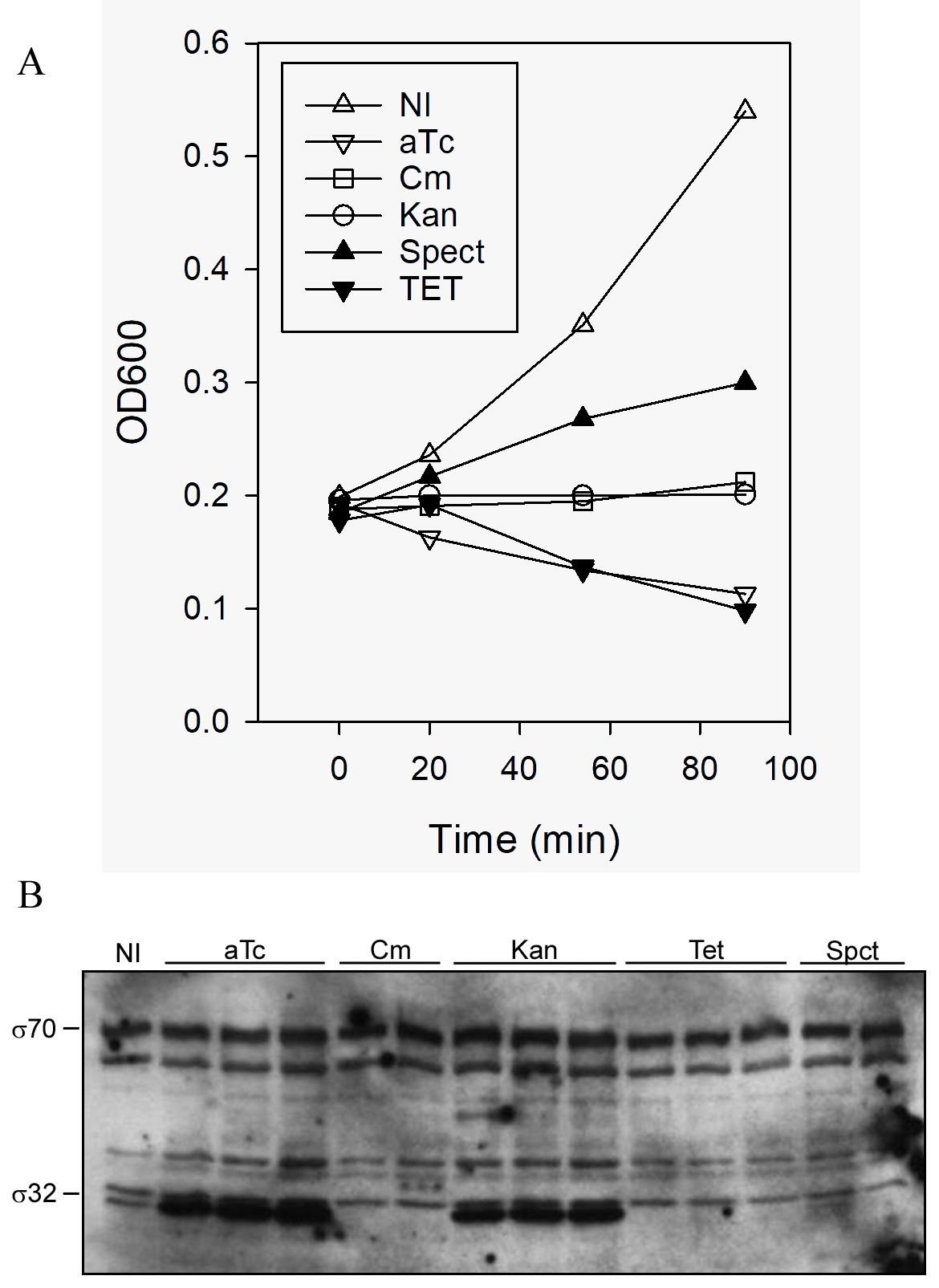

Supplement: S2 Fig — A, a culture of BL21 cells transformed by pARF48 was divided at early log phase and treated with either aTc (0.2 μg/ml) to induce ARF48 expression, or with Cm, Kan, Tc or Spectinomycin (Spct) at 30, 25, 50 or 20 μg/ml, respectively. An aliquot of culture also was left untreated (NI) for comparison. Cultures were then incubated at 37°C with shaking, and cell growth was monitored by measuring culture optical density at 600 nm (OD600) using a microplate reader. B, samples of each culture were collected at 20, 60 and 90 min after treatment, and cells were lysed in 2X SDS-PAGE sample buffer at a concentration of 1.2 OD600 units per ml. Equal volumes of each sample were then analyzed by Western blotting, using antibodies against σ32 and σ70. Only the 20 min and 60 min samples of the Spectinomycin-treated culture were analyzed. (TIF) [file pone.0258794.s002.tif]

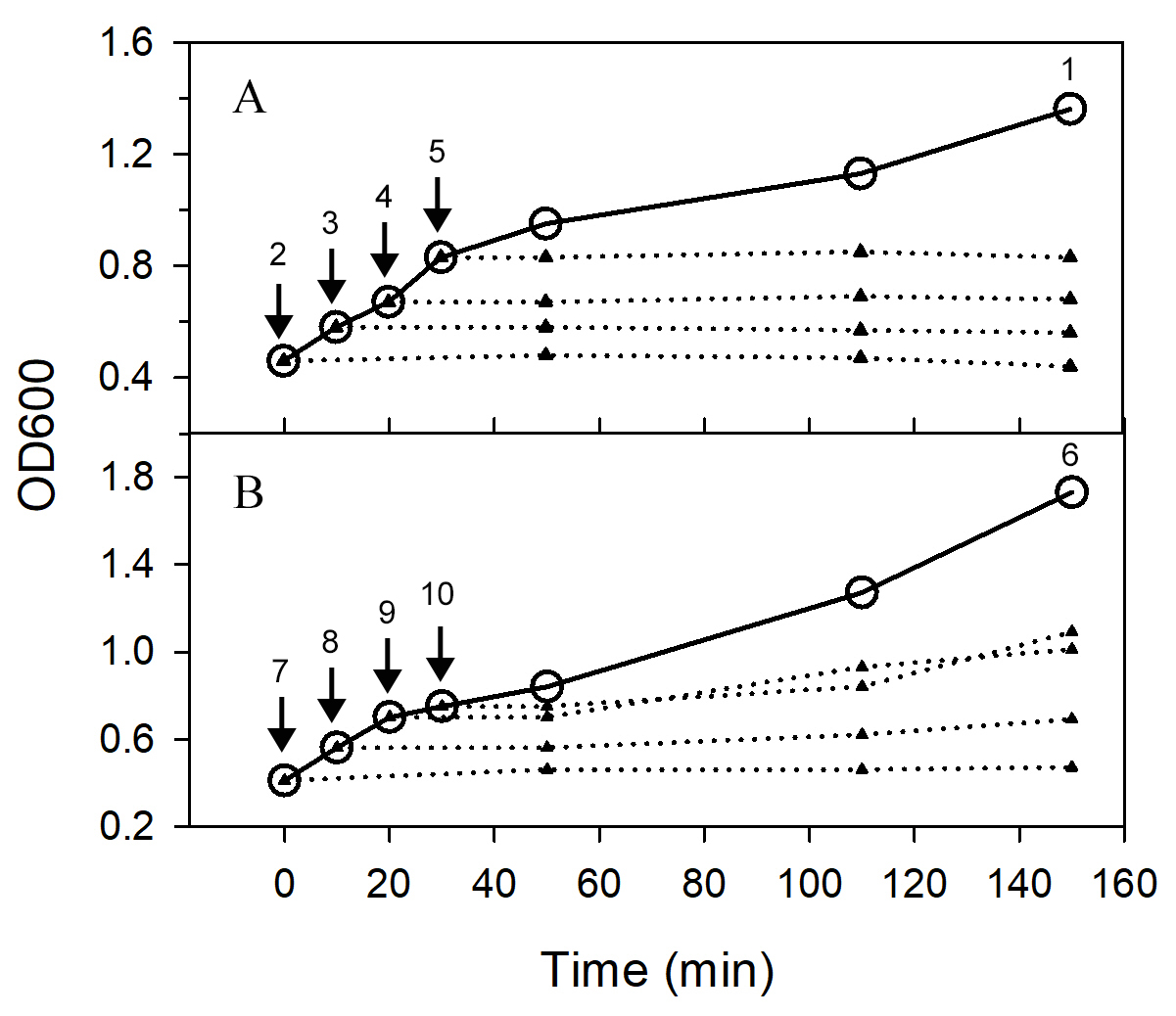

Supplement: S3 Fig — E. coli BL21DE3 cells were co-transformed with pARF48, a ColE1 replicon, and pACYCDuet-RpoA or pACYCDuet-RpoB, which are p15A-based replicons from a different plasmid incompatibility group that contain genes coding for the E. coli RpoA and RpoB proteins cloned behind the IPTG-inducible T7/lac promoter. Early log phase cultures of the co-transformants were treated with IPTG at time 0 to induce expression of RpoA or RpoB (panels A and B, respectively), and cell growth was then monitored by measuring culture OD600 (solid lines with open circles). At 0, 10, 20 or 30 min later, samples of culture were removed and treated with aTc to induce ARF48 expression (numbered arrows). Growth of the aTc-treated cultures also was monitored as above (dotted lines with filled symbols). (TIF) [file pone.0258794.s003.tif]

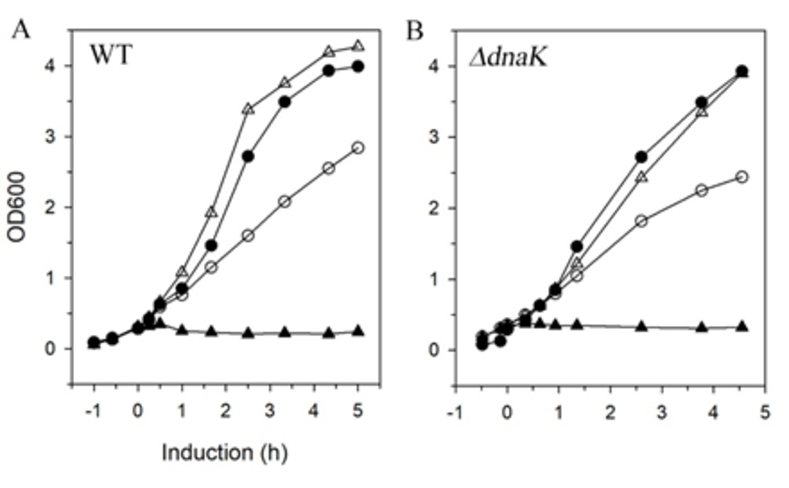

Supplement: S4 Fig — A, growth of E. coli BL21 cells (which contain wild-type DnaK) following expression of ARF48 or the ARF-NR and ARF-DA variants. △, non-induced control; ▲, ARF48; ○, ARF-NR; ●, ARF-DA. B, same experiment as A, but proteins were expressed in a mutant E. coli strain that lacks the DnaK gene (ΔdnaK) [33]. The results shown are representative of 3 independent experiments. (TIF) [file pone.0258794.s004.tif]

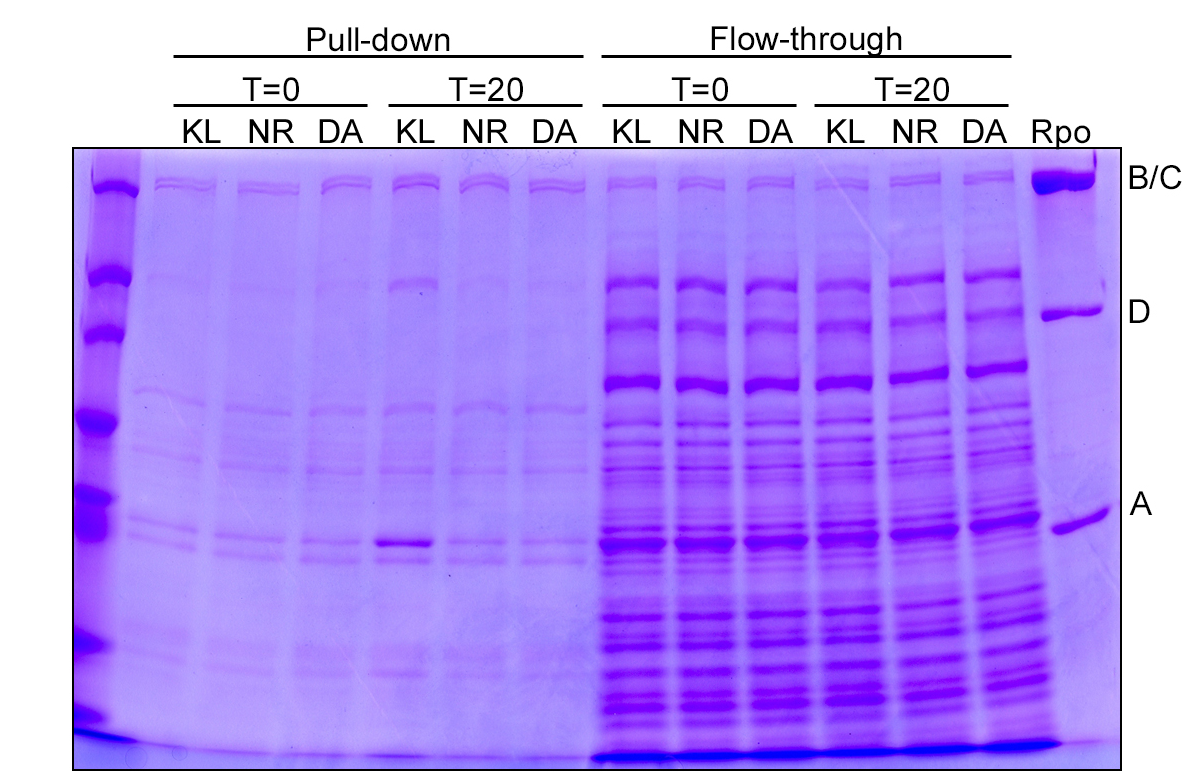

Supplement: S5 Fig — An exact duplicate SDS-polyacrylamide gel of the Western blot shown in Fig 5 was stained with Coomassie blue. A sample of purified RNA polymerase was loaded in the last lane (labeled rpo). Bands corresponding to the RNA polymerase α, β, β′, and σ70 subunits are labeled (A, B/C, and D, respectively). (TIF) [file pone.0258794.s005.tif]

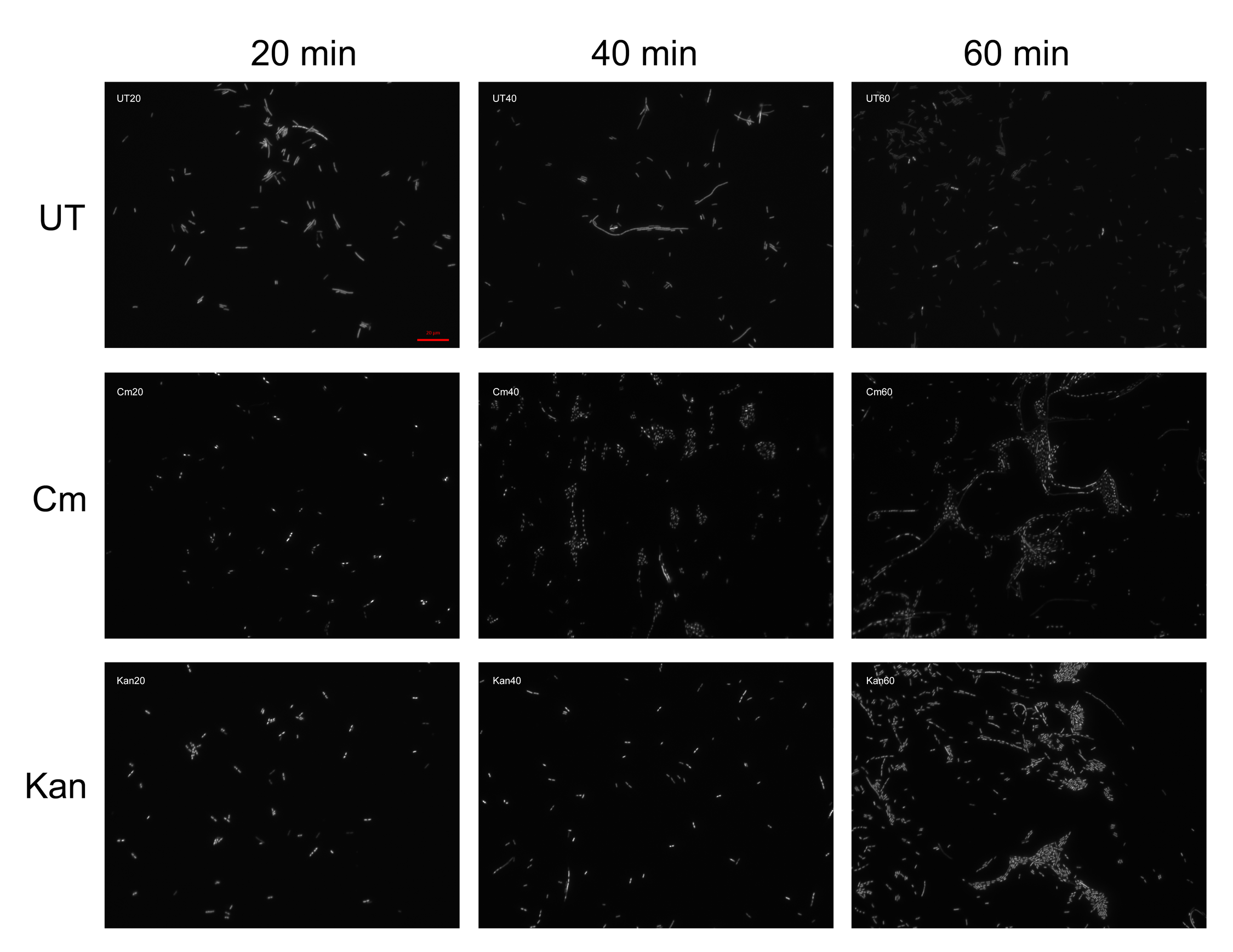

Supplement: S6 Fig — A culture of E. coli BL21 cells growing in LB broth at 37°C was divided and treated with either Cm (30 μg/ml) or Kan (50 μg/ml) at early log phase. A culture sample also was left untreated (UT) for comparison. Cell samples were then collected after further incubation for 20, 40 or 60 min, washed with PBS, stained with H33342, and mounted on glass slides under agarose pads [17] for fluorescence microscopy using a 40X oil immersion lens. The exposure time for each image was automatically set by microscope software. Scale bar = 20 μm. (TIF) [file pone.0258794.s006.tif]

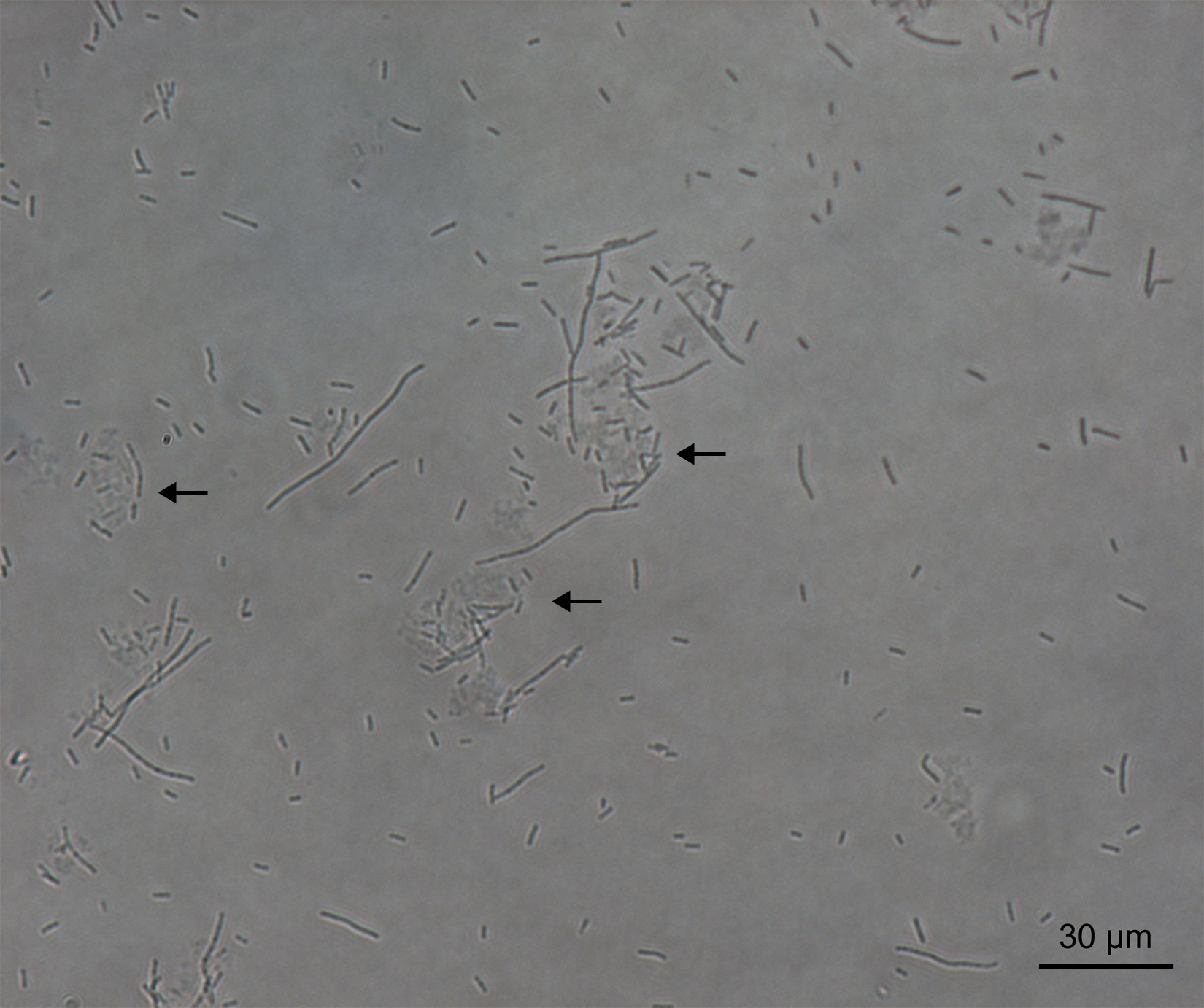

Supplement: S7 Fig — ARF48 expression was induced at early log phase in E. coli BL21 cultures growing either in standard LB broth, which contains 0.17 M NaCl or in modified LB broth lacking NaCl (see Fig 7). 2 h after induction, cell samples from induced and non-induced cultures were washed in PBS, fixed in 4% paraformaldehyde, and then examined by brightfield microscopy. When cell suspensions were mounted under standard glass coverslides, cells from both induced cultures formed cell aggregates (arrows), whereas cells from non-induced cultures did not aggregate. By contrast, when cell suspensions were mounted under agarose pads, which blocks diffusion of cells on the microscope slide, cell aggregation was not observed. Similarly, cell aggregation was not observed when cells from induced cultures were suspended in salt-free buffer (10 mM phosphate pH 7.4) and mounted under standard glass coverslides. (TIF) [file pone.0258794.s007.tif]
