# Supplementary material for: A polypeptide model for toxic aberrant proteins induced by aminoglycoside antibiotics
Source: PLoS One. 2022 Apr 29;17(4):e0258794. doi: 10.1371/journal.pone.0258794 (PMC9053816; doi:10.1371/journal.pone.0258794)

original blot for Fig 1D

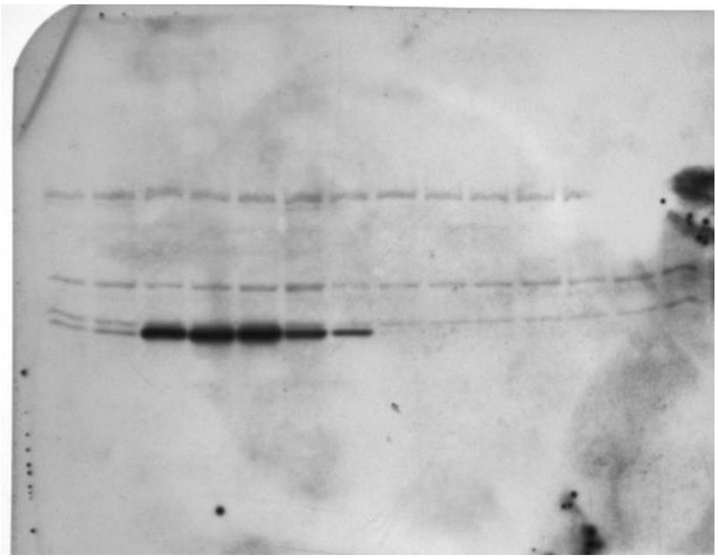

orginal blot for Fig 1E

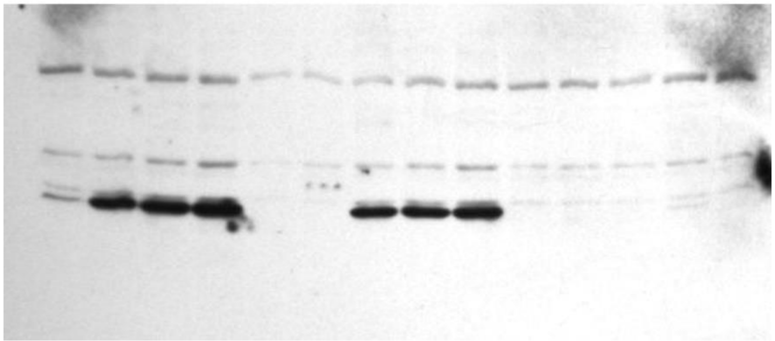

original gel for Fig 3B

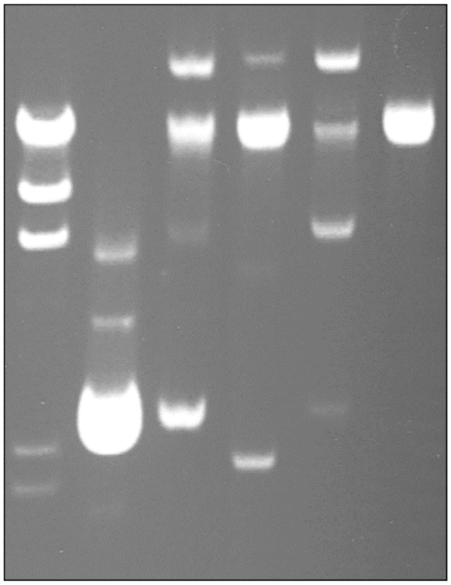

original gel for Fig 3C

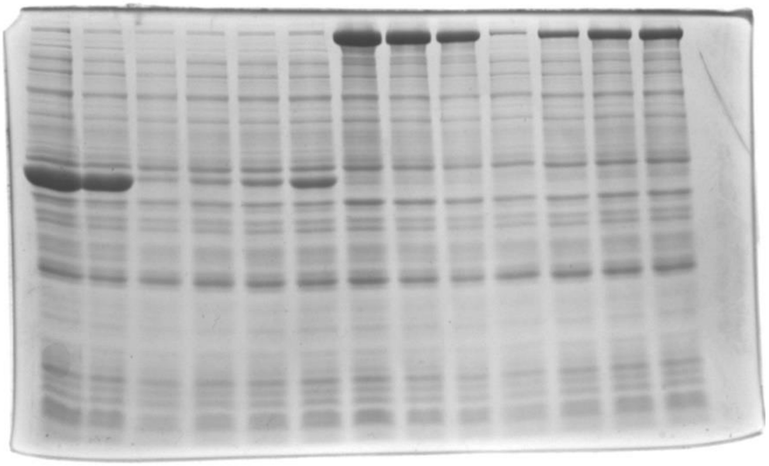

original blot for Fig 4B_sigma 32 probe

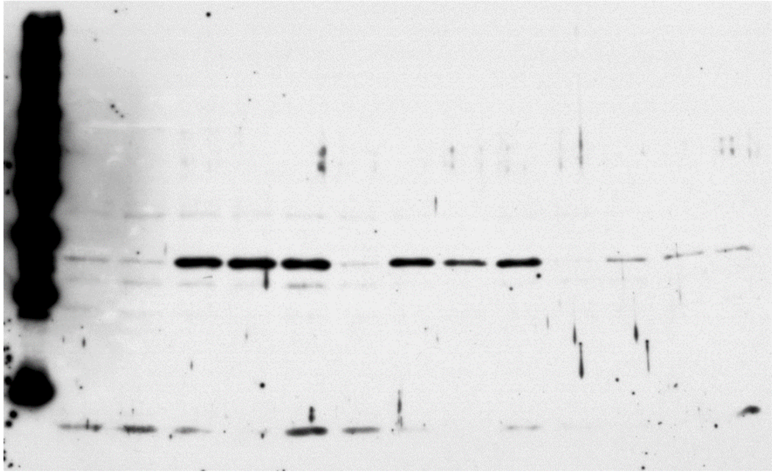

original blot for Fig 4B_sigma 70 probe

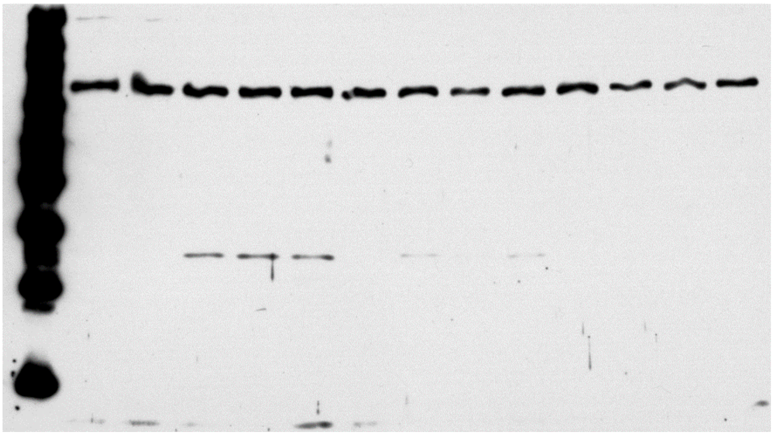

original blot for Fig 4C_GroEL probe

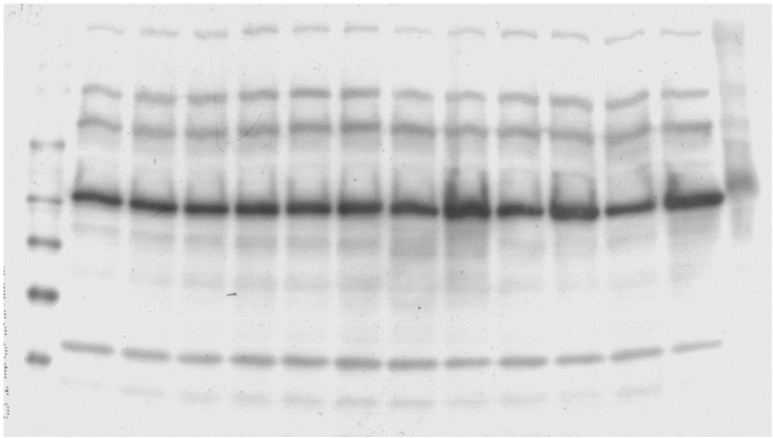

original blot for Fig 4C_DnaJ probe
(GroEL blot re-probed with anti-DnaJ antibody)

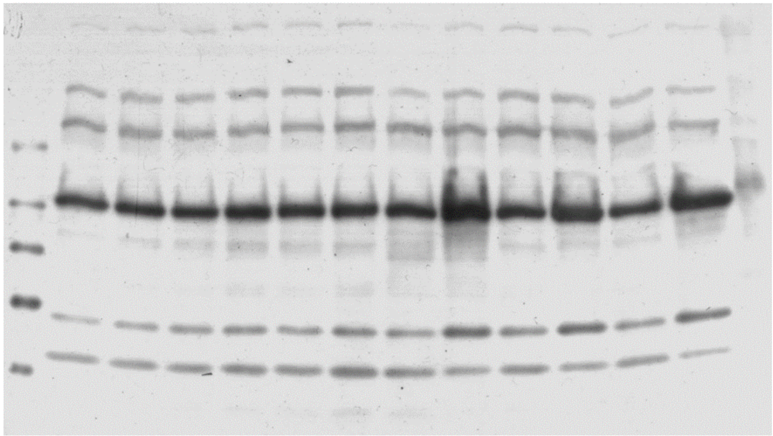

original blot for Fig 4C_DnaK probe

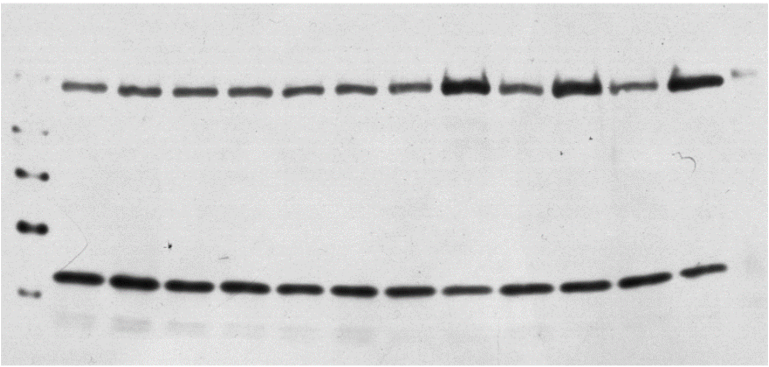

original blot for Fig 5_sigma 70 probe

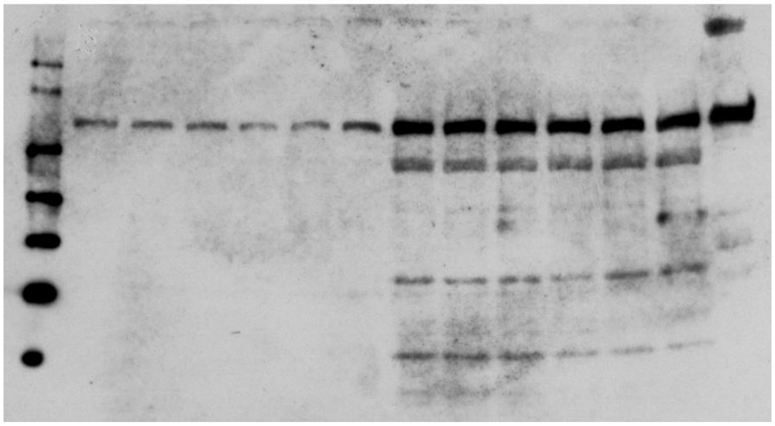

original blot for Fig 5_sigma 32 probe
(sigma 70 blot re-probed with anti-sigma 32 antibody)

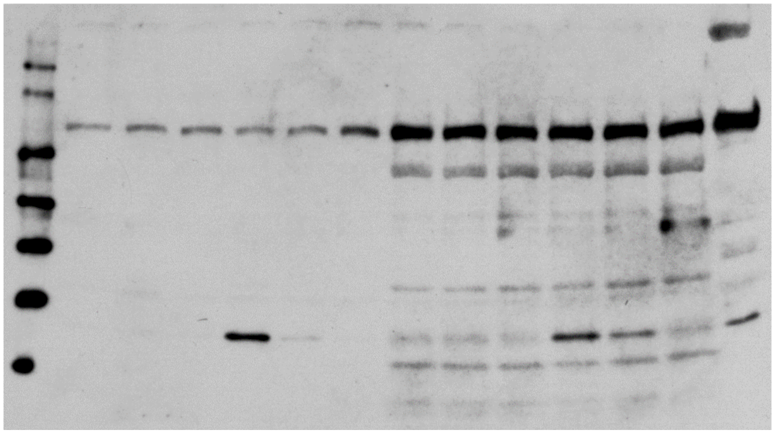

original gel for S5 Fig (supplemental Fig 5)

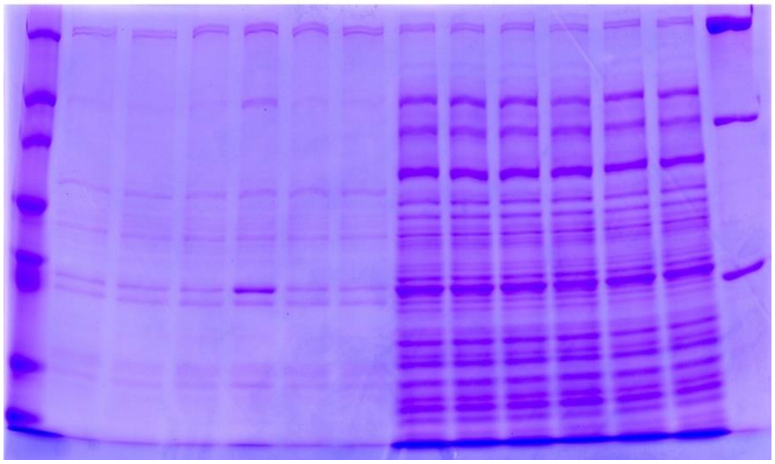

Supplement: S1 Raw images — (PDF) [file pone.0258794.s008.pdf]
